# An environmental monitoring data sharing scheme based on attribute encryption in cloud-fog computing

**DOI:** 10.1371/journal.pone.0258062

**Published:** 2021-09-30

**Authors:** Xiaodong Yang, Wanting Xi, Aijia Chen, Caifen Wang

**Affiliations:** 1 Department of Computer Science and Engineering, Northwest Normal University, Lanzhou, Gansu, China; 2 Department of Big Data and Internet, Shenzhen Technology University, Shenzhen, Guangdong, China; University College of Engineering Tindivanam, INDIA

## Abstract

Environmental monitoring plays a vital role in environmental protection, especially for the management and conservation of natural resources. However, environmental monitoring data is usually difficult to resist malicious attacks because it is transmitted in an open and insecure channel. In our paper, a new data sharing scheme is proposed by using attribute-based encryption, identity-based signature and cloud computing technology to meet the requirements of confidentiality, integrity, verifiability, and unforgerability of environmental monitoring data. The monitoring equipment encrypts the monitored environmental data and uploads it to the environmental cloud server. Then, monitoring users can request access to the environmental cloud server. If the monitoring user meets the access policy, the plaintext is finally obtained through the fog node decryption. Our proposal mainly uses attribute-based encryption technology to realize the privacy protection and fine-grained access control of monitoring data. The integrity and unforgeability of the monitoring data are ensured by the digital signature. In addition, outsourcing computing technology saves the computing overhead of monitoring equipment and monitoring users. The security analysis illustrates that our proposal can achieve security purposes. Finally, the performance of our proposal and related schemes is evaluated from the aspects of communication overhead and computing overhead. The results indicate that our proposal is secure and efficient in environmental monitoring.

## Introduction

With vigorous economic and social development, population growth and industrial development are accompanied by the production of a large number of waste pollutants. These pollutants seriously endanger the environment and affect human health and survival. Therefore, environmental construction has received increasing attention from human society. Environmental monitoring is a critical link in environmental protection. It plays an essential role in discovering environmental problems and providing data references for formulating environmental policies [1].

The emergence of environmental monitoring technology perfectly solves the problem that manual measurement cannot be made in harsh environments, such as extremely cold weather and nuclear radiation areas. However, while the massive application of environmental monitoring equipment brings convenience, the transmission and sharing of massive monitoring data also imply huge data privacy security risks [2]. Because environmental monitoring data is transmitted in an insecure open channel, it is usually difficult to resist various malicious attacks. At the same time, environmental monitoring data is also a lucrative target for hackers or criminals. Criminals may deliberately interfere with the normal development of environmental monitoring activities, intentionally tampering with the monitoring data, resulting in distortion of the monitoring data [3]. For example, evil enterprises may hire hackers to tamper with environmental monitoring data driven by their profits maliciously, and continue to discharge sewage in excess of the standard to harm the environment. In addition, severe distortion of environmental monitoring data will reduce the credibility of the government, and will also affect the correctness of decision-making by environmental administrative departments.

Environmental monitoring has attracted widespread attention from the industry and academia, especially the privacy protection and security sharing of monitoring data [4]. First, environmental monitoring data may contain highly private information of individuals and companies. For example, property owners identified near sources of harmful pollution that could experience decreased property values. Thus, privacy protection is the protection of corporate reputation and interests. Second, only real and complete environmental monitoring data can guarantee the implementation of environmental protection activities. The forged or modified monitoring data can lead to faulty decisions by the Environmental Protection Agency. In addition, the sharing of monitoring data improves the efficiency of environmental monitoring and enhances the mobility of data between environmental protection departments in different regions.

In response to these problems, attribute-based encryption technology [5] is proposed for the privacy protection of environmental monitoring data. Attribute-based encryption can easily realize one-to-many data secure transmission, which is very suitable for environmental monitoring. Attribute-based encryption realizes the fine-grained access control and data confidentiality by embedding access policy into ciphertext [6]. The access policy is a specific attributes collection, and the decryption succeeds only if the monitoring user’s attributes match the access policy [7]. Although these works provide useful schemes for the privacy protection of environmental monitoring data, there is still a problem of low practicability. In the practical application of environmental monitoring, attribute-based encryption will inevitably have user permissions change or attribute expiration. Therefore, the system also needs to revoke users, update and delete attributes, and other operations according to actual requirements [8]. However, none of the above schemes can guarantee the authenticity and integrity of the data collected by the monitoring equipment before encryption. The digital signature can solve this problem well, and it can achieve the authenticity and unforgeability of monitoring data [9–11]. Once the monitoring equipment is compromised by criminals, the equipment may transmit the tampered error monitoring data. The relevant environmental department can confirm the authenticity of the data through signature verification to trace back which equipment transmitted the wrong data [12]. Even though these works have achieved the purpose of protecting the privacy of monitoring data, there is still a problem that the monitoring equipment and users cannot afford a huge computing overhead, owing to the huge amount of calculations in attribute-based encryption. It is also worth considering how to secure the sharing of monitoring data.

**Fig 1:**
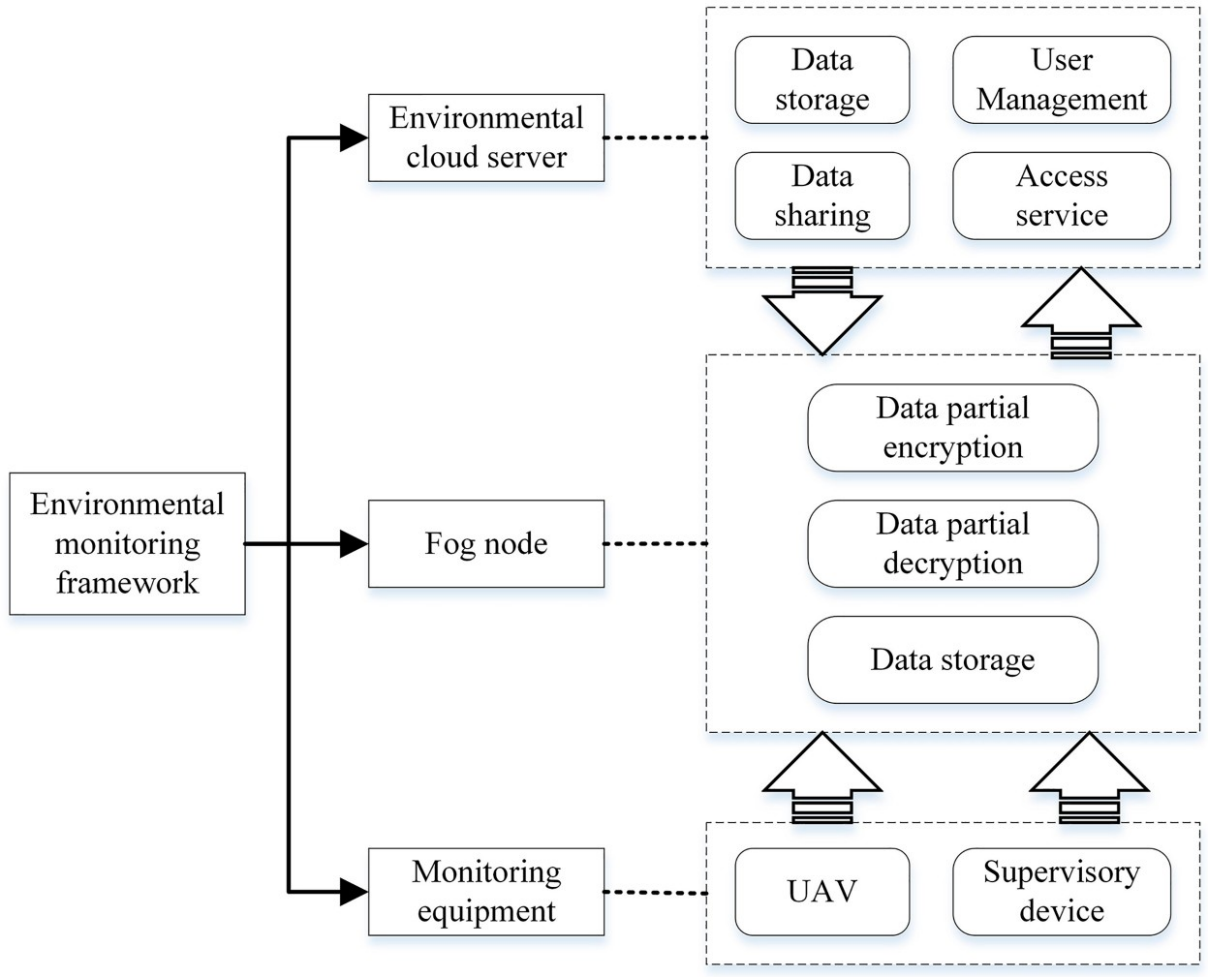
A three-layer framework of environmental monitoring.

Cloud computing [13] and fog computing [14] are new computing models based on sharing, and are forward-looking solutions to the security problem of environmental monitoring data sharing. Cloud-fog computing with massive computing resources and memory capacity is very suitable for the management, sharing, and storage of environmental monitoring data [15]. The combination of cloud-fog computing and terminal equipment forms an environmental monitoring system under the three-layer structure of cloud, fog, and terminal shown as Fig 1. As an extension of cloud computing, fog computing migrates computing to fog nodes closer to terminals, which effectively solves the problem of high latency between cloud servers and user terminals, but users still have to bear a huge computing overhead [16]. The outsourcing technology under cloud-fog computing can reduce the user’s computing overhead [17]. It outsources a mass of encryption and decryption operations to cloud servers and fog nodes, and users only need a few simple calculations to obtain plaintext data [18]. Although attribute-based encryption and cloud-fog computing technology have a series of advantages in privacy protection and secure sharing of environmental monitoring data, we still face the following challenges: 1) How to efficiently achieve the privacy protection and secure sharing of environmental monitoring data based on cloud-fog computing? 2) How to ensure that only legitimate monitoring users can access monitoring data? 3) How to prevent the occurrence of collusion to maintain the system running safely and normally?

To meet these challenges, we propose a new secure and efficient environmental monitoring data sharing scheme based on cloud-fog computing. The scheme combines attribute-based encryption, digital signature, and cloud-fog computing technology to realize the privacy protection and secure sharing of environmental monitoring data. Outsourcing computing in cloud-fog environment reduces the computing burden of monitoring users and solves the time-consuming and inefficient problems of most attribute-based encryption schemes [19]. In addition, our scheme uses a combination of attribute-based encryption and revocation mechanism to ensure that only legitimate monitoring users can access monitoring data. We have also introduced a multi-authorization mechanism to ensure that there is no excessive Environmental Protection Agency authority collude with illegal users [20, 21]. In summary, the main contributing factors of our proposal are as follows:

- We propose a secure and efficient environmental monitoring data sharing scheme. The scheme combines attribute-based encryption, digital signature, and cloud-fog computing technology to realize the confidentiality, intergrity, verifiability, and unforgeability of environmental monitoring data, which ensures the privacy protection and secure sharing of environmental monitoring data.
- We adopt computing outsourcing technology to deal with the lack of efficiency in the sharing of environmental monitoring data. A huge amount of calculations?are outsourced to fog computing nodes with massive computing resources, which not only reduces the computing overhead but also ensures the efficiency of the system.
- We design a revocation method to ensure the flexibility of our proposal. Fine-grained revocation in attribute-based encryption enables only legitimate users to access the monitoring data, and illegal users will be denied access by revoked permissions.
- We introduce a distributed multi-authorization mechanism. To prevent the EPA from spamming keys and colluding with illegal users due to excessive authority.
- We analyze the security and performance of the scheme. Advanced schemes are used to evaluate and compare with our proposal, which proves that our proposal is secure and efficient.

The rest sections of this paper are arranged as follows. Section 2 gives the key technologies and tools prepared for our scheme. Section 3 introduces the system model and the practical application scenarios of each entity. Later, section 4 shows the construction and implementation of the specific scheme. Then, the security analysis is provided in Section 5. Furthermore, the performance evaluation process and the results of our proposal are presented in Section 6. In the end we conclude the whole paper and outline the direction of future work in section 7.

## Related work

In this section, we will discuss the related work focusing on the privacy protection and secure sharing of monitoring data and the technical basis supporting this work.

### Smart environmental monitoring system

Environmental monitoring includes proper planning and management of disasters, control of different pollution, and effective response to challenges arising from unhealthy external conditions. Environmental monitoring mainly includes soil monitoring (SM) [22], air quality monitoring (AQM) [23], water quality monitoring (WQM) [24] and radiation monitoring (RM) [25] applied to different fields. Soil quality, air quality, water pollution, and radiation pollution are the factors that pose real challenges to the environment. Appropriate monitoring is necessary so that the world can achieve sustainable growth by maintaining a healthy society. In recent years, with the development of the Internet of things (IoT) and modern sensors, environmental monitoring has been transformed into smart environmental monitoring (SEM) [26]. However, environmental monitoring infrastructures such as sensors are easy to be attacked by illegal users, and the monitoring data transmitted by them are also vulnerable to leakage and tampering. Therefore, it is very important to encrypt the monitoring data. The author of literature [27] proposed the use of wireless sensor networks in rural areas to monitor environmental factors such as temperature, humidity, and solar radiation. However, this only guarantees the availability of data and does not mention the security issues of data communication, and the program has a 20 percent data loss rate during transmission. Simitha et al. [28] used applications based on wireless sensor networks to monitor water quality in urban environments. This proposal uses a low-energy system to increase its autonomy, but it does not mention the basic aspects of security, which can not ensure the integrity and confidentiality of monitoring data. In the literature [29], Potter et al. collected monitoring data such as water temperature, dissolved oxygen, and pH value from sensor nodes deployed in the water stream. However, the system is designed to operate in a threat-free environment without using measures and protocols to protect data security; the exponential expansion of the Internet of Things increases the risk of sensor attack, so we must consider the data security of environmental monitoring.

### The technical basis of cryptography

Data security runs through the entire process of data collection, transmission, storage, and application. It can be said that all stages of the monitoring data life cycle are faced with security risks. Cryptography can provide theoretical and technical support for the privacy protection and secure sharing of monitoring data. Sahai and Waters [30] first proposed the concept of attribute-based encryption. They introduced attribute set and access policy to encrypt data, which can protect data confidentiality and realize access control at the same time. Literature [31] adopts an attribute-based encryption algorithm based on ciphertext policy, which also provides a fine-grained access control method on the basis of protecting the confidentiality and integrity of data. However, the scheme is a single authorization scheme, which can not prevent the collusion between the authority center and users or the abuse of keys. Azees et al. [32] proposed an efficient group key distribution scheme for secure group communication based on bilinear pairing. The proposed CEKD scheme provides good performance in terms of computational cost, but single authorization may lead to a single point of failure for a trusted institution. To solve the security problems caused by a single authorization center, Ruj et al. [33] proposed an attribute-based encryption scheme with multiple authorizations that can be revoked by users. However, their scheme needs to re-provide ciphertext components to users who have not been revoked, which increases the communication cost of the system. The above literature only consider the security issues of user-side data, and do not consider the security issues that may exist on the data collection side. Therefore, we embed digital signature technology into the environmental monitoring data collection process to ensure the integrity and unforgeability of the data. At the same time, identity verification is considered to be the first line of defense against malicious users who may leak and tamper with data by impersonating legitimate users without verification [34]. Moreover, revoking malicious users is very necessary for other users who continue to interact in the system. Vijaykumar et al. [35] proposed an anonymous authentication technology based on the digital signature, which can prevent malicious users from entering the system and provide data integrity for message transmission. At the same time, the revocation mechanism is introduced to revoke malicious users during disputes and misconduct.

### Cloud-fog computing technology

Google puts forward the concept of “cloud computing” for the first time [36]. Cloud computing provides many enterprises and individuals with a brand-new service model and a better service platform. However, due to the centralized distribution of cloud servers, the delay between cloud servers and the user equipment is relatively high. The emergence of fog computing has made up for this shortcoming, and fog computing is an extension of cloud computing. Cloud computing stores and transmits large amounts of data. As a connection between cloud computing and IoT terminal devices, fog computing shares part of the computing work of cloud computing and terminal devices, and its low latency also speeds up data transmission [37]. Alrawais [38] and others proposed an attribute-based privacy protection scheme in fog computing, which saves the network overhead of the cloud platform and improves the efficiency of access control. However, they did not consider the large computing overhead of the Internet of Things devices with limited resources. Zuo et al. [39] proposed an ABE scheme for outsourcing decryption that supports fog computing, which is secure against chosen-ciphertext attacks. However, the scheme only outsources the decryption operation to the fog node, and does not consider the huge overhead of encryption operation that still needs to be borne by the terminal IoTs equipment. Li et al. [40] proposed a scheme to support verifiable outsourcing encryption and decryption. In the scheme, they also outsourced the encryption operation on the data owner’s side.

## Preliminaries

In this section, we discuss cryptography knowledge and tools which support environmental monitoring data privacy protection and secure sharing.

### Bilinear map

Assume that *P* is a large prime number, *G*_0_ and *G*_*T*_ both are the cyclic group of order *P*, and a generator of the cyclic group *G*_0_ is *g*. Then the bilinear mapping should have the following three characteristics.

- Bilinear: For any *a*, *b* ∈ *Z*_*P*_ and *g*, *h* ∈ *G*_0_, it has *e*(*g*^*a*^, *h*^*b*^) = *e*(*g*, *h*)^*ab*^.
- Non-degenerate: There exists *g* ∈ *G*_0_, such that *e*(*g*, *g*) ≠ 1, where the unit element of *G*_*T*_ is 1.
- Computability: For any *g*, *h* ∈ *G*_0_, there is an efficient computation *e*(*g*, *h*).

### Difficult problems

Decisional Bilinear Diffie-Hellman Problem (DBDH): *G*_0_ is a cyclic group of prime order *P* and *g* is the generator of the cyclic group *G*_0_, the bilinear mapping is *e*: *G*_0_ × *G*_0_ → *G*_*T*_. Choose random number *a*, *b*, *c* ∈ *Z*_*p*_. For the polynomial time algorithm, when given (*g*, *g*^*a*^, *g*^*b*^, *g*^*c*^), the adversary cannot distinguish *e*(*g*, *g*)^*abc*^ and the random number *R* ∈ *G*_*T*_, then the DBDH hypothesis is valid.

Computational Diffie-Hellman Problem (CDH): *G*_0_ is a cyclic group with prime order *P*. Let *a*, *b* ∈ *Z*_*p*_ be random elements in *G*_0_ and give the triples (*P*, *aP*, *bP*), calculate *abP* ∈ *G*_0_ is difficult.

## System model

**Fig 2:**
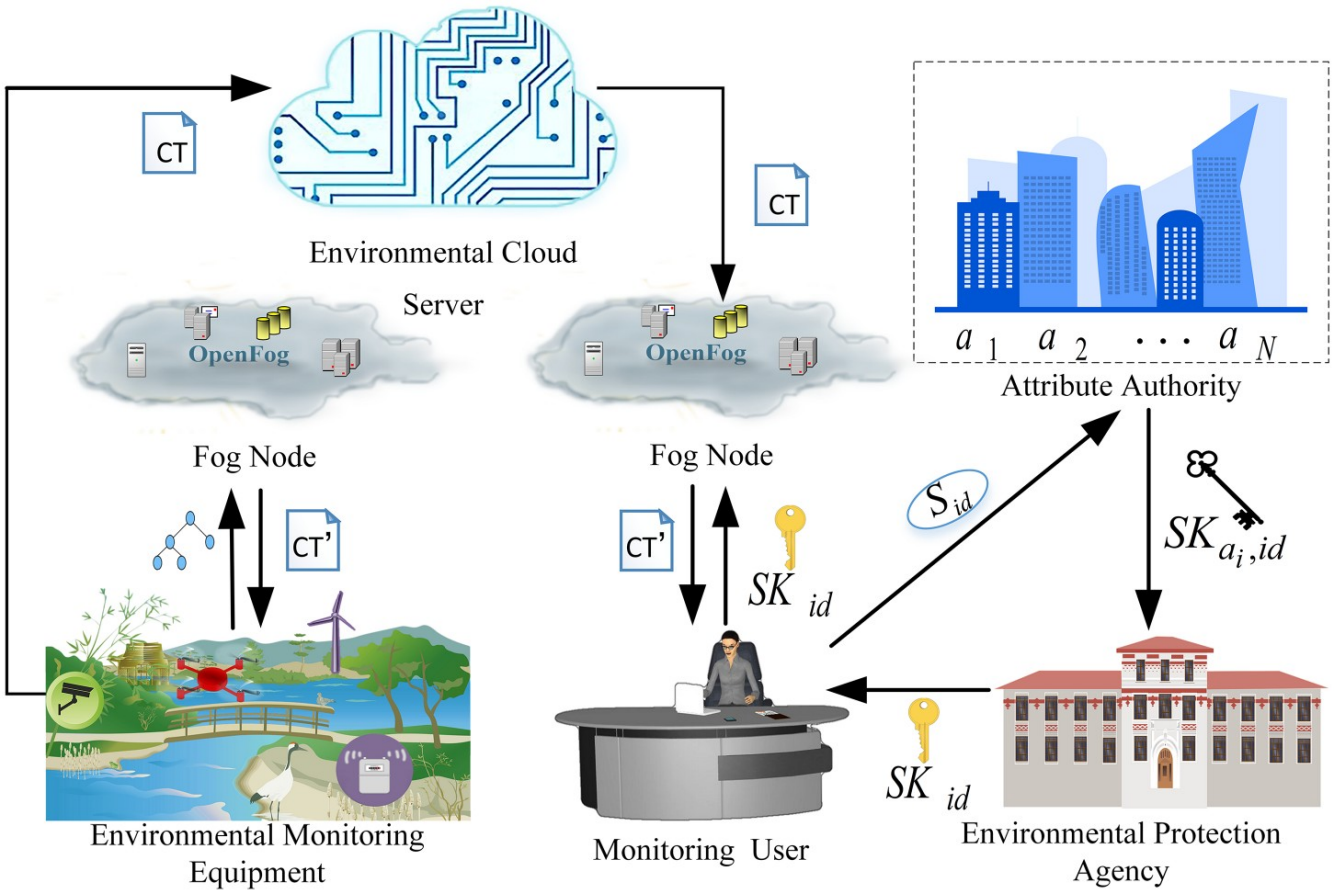
System model. (The source of the components of Fig 2 is described in the Supporting information).

The system model proposed in this paper mainly consists of six entities: environmental cloud server, fog computing node, environmental monitoring equipment, monitoring user, environmental protection agency and attribute authority, shown as Fig 2.

- Environmental Cloud Server (ECS): The environmental protection cloud server is mainly responsible for the management and storage of the monitoring data. Due to the security risk of leaking private data, the environmental cloud server is semi-trusted. When the fog node uploads the signature to the ECS, the ECS is responsible for verifying whether the signature is valid. When an attribute revocation event occurs, the ciphertext can be updated to prevent the revoked user’s access.
- Fog Node (FN): The Fog computing node is mainly responsible for environmental monitoring data storage and part of encryption and decryption calculations. The monitoring equipment outsources partial encryption calculation to the fog node. And the monitoring users outsource partial decryption calculation to the FN, to mitigate the computing burden of the terminal devices. When the monitoring equipment uploads a partial signature to the fog node, the FN is responsible for verifying whether the partial signature is valid. The fog computing node is also semi-trusted and communicates with the ECS, EME, and MU.
- Environmental Monitoring Equipment (EME): Environmental monitoring equipment mainly refers to the Internet of things equipment with limited resources to collect monitoring data, such as supervisory devices, drones, sensors, etc. The massive amounts of environmental monitoring data encrypted by them need to be uploaded to the ECS for storage.
- Monitoring User (MU): Environmental monitoring users are accessing users or devices who want to obtain environmental monitoring data information. They send access requests to the environmental cloud server. Only legitimate users who meet the access policy could access the monitoring data correctly, while illegal users are denied access because they cannot correctly match the access policy.
- Environmental Protection Agency (EPA): As the central authority, the EPA only participates in the system initialization phase. The EPA is mainly responsible for the release of the master key and public parameters of entire system. The Environmental Protection Agency is completely trusted.
- Attribute Authority (AA): The generation and distribution of the user’s attribute private key is mainly responsible by the AA. When the MU sends their attributes to AA to request the key generation, the AA generates the attribute private key from the user’s attributes and transfers it to the EPA.

## Proposed scheme

In this section, we begin with an overview of a secure and efficient environmental monitoring data sharing scheme. After that, we will elaborate on the specific construction process of the scheme.

### Overview

**Fig 3:**
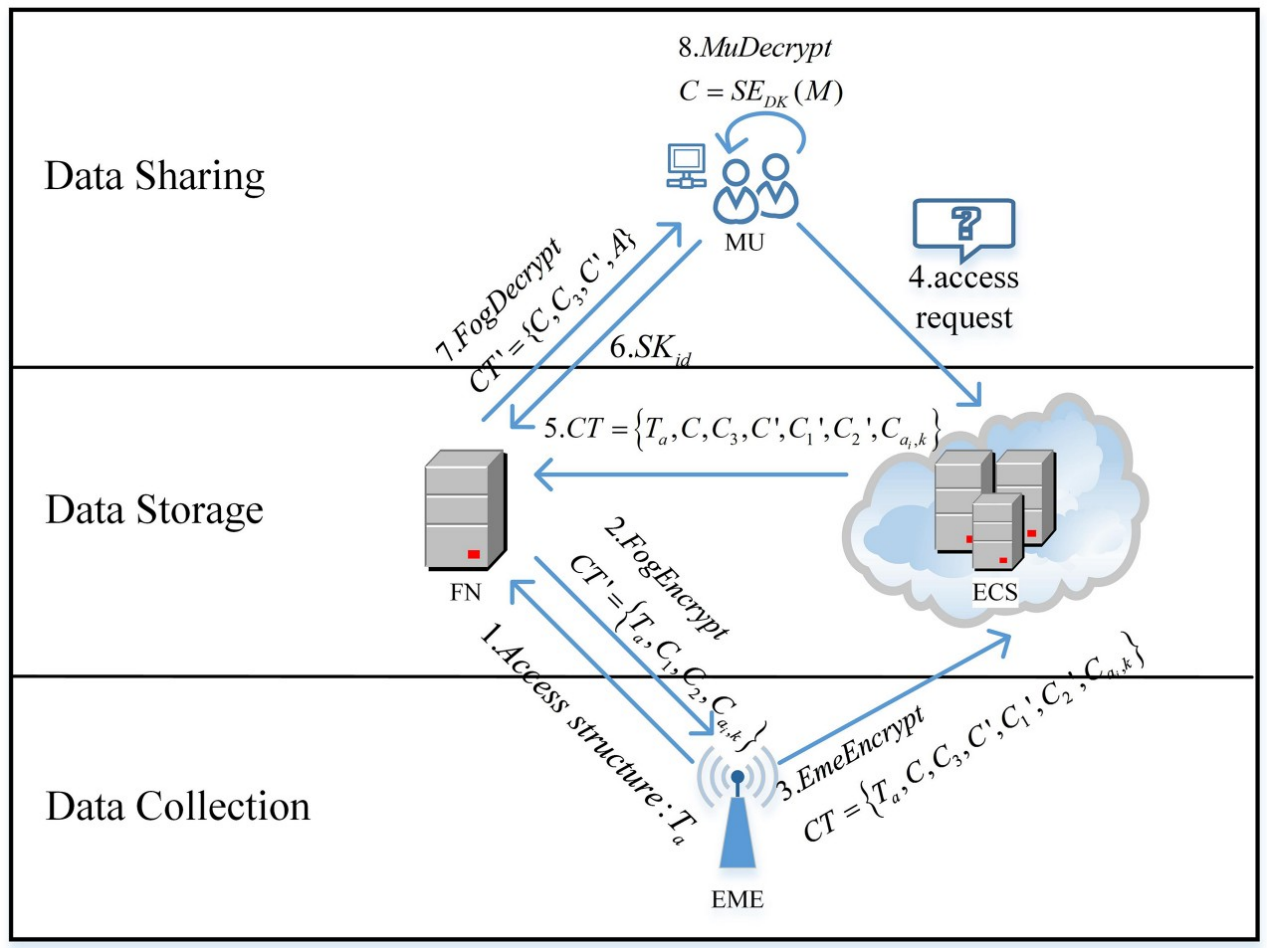
Process overview.

Environmental monitoring typically uses monitoring equipment that is easy to deploy and less expensive to collect data. However, monitoring equipment has limited computing resources and limited storage capacity, so it is necessary to rely on the environmental cloud servers and fog nodes to manage and store monitoring data. Monitoring users communicate with fog nodes and environmental cloud server to obtain monitoring data. The monitoring equipment collects data from the monitoring environment and attaches a signature to it. The data will later be transmitted to the fog computing node for encryption calculation. Then upload the monitoring data to the environmental cloud servers for storage, which provides scientific evidence for environmental management, contamination control, and environmental planning. At the same time, the environmental cloud server also facilitates users of various environmental protection departments and environmental monitoring stations to share and access the monitoring data. The monitoring user submits a data access request, and the environmental cloud server responds by sending the ciphertext to the fog node for partial decryption. And then the user can restore part of the ciphertext to plaintext data with very few calculations. The overall process of our proposal is shown in Fig 3, which is specifically structured at the levels of data collection, data storage, and data sharing respectively.

### Construction

Our scheme is designed mainly to provide secure data sharing on public channel, divided into five phases: system initialization, key generation, monitoring data storage, monitoring data sharing, and revocation.

#### System initialization

The system initialization can be divided into two parts: EPA setup and AA setup.

1. EPA setup
  Environmental Protection Agency generates public parameter and master key, and assigns a unique identity value to monitoring equipment and each monitoring user. The EPA picks λ as security parameters to generate a bilinear group *G*_0_ of prime order *P*. The EPA selects bilinear mapping *e*: *G*_0_ × *G*_0_ → *G*_*T*_, and selects three collision-resistant security hash functions *H*_1_: {0, 1}* × *G*_0_ → *Z*_*p*_, *H*_2_: {0, 1}* → *Z*_*p*_, *H*_3_: {0, 1}* → *G*_0_. Assuming that there are j attributes in the whole system and the number of attribute authorities is N, the attribute set is specified?as *S* = {*s*_1_, *s*_2_, *s*_3_, ⋯, *s*_*j*_} and attribute authority set is specified as *A* = {*a*_1_, *a*_2_, *a*_3_, ⋯, *a*_*N*_}. The EPA selects random number *α*, *β* ∈ *Z*_*p*_, *h* ∈ *G*_0_, then the master key and public parameter generated as *MK* = {*g*^*α*^, *β*}, *Params* = {*G*_0_, *g*, *h*, *g*^*α*^, *g*^*β*^, *e*(*g*, *g*)^*αβ*^, *S*, *A*, *H*_1_, *H*_2_, *H*_3_}. The EPA assigns a unique identity value *id* to each monitoring equipment and each monitoring user, and adds it into the equipment and user list *U*.
2. AA setup
  There can be multiple attribute authority, and each attribute authority manages different attribute sets. The attribute authority *a*_*i*_ generates its key pair according to the attribute set $S_{a_{i}}$ and public parameter *Params*. The attribute authority selects the probabilistic generation function *AASetup*, which inputs the public parameter *Params* and the attribute set $S_{a_{i}}$ authorized by *a*_*i*_, where $a_{i}\in A,S_{a_{i}}\subseteq S$. The attribute set $S_{a_{i}}$ authorized by each attribute authority is different from each other, that is to say $S_{a_{1}}\cap S_{a_{2}}\cdots \cap S_{a_{N}}=\emptyset$, the collection of each authority’s attribute sets is *S*. AA select random number *t*, *q* ∈ *Z*_*p*_, the function outputs the attribute authority public and private keys as $APK_{a_{i}}=\left\{g^{t_{a_{i}}},{\left\{g^{q_{a_{i},k}\cdot t_{a_{i}}}\right\}}_{1\leq k\leq |S_{a_{i}}|}\right\}$, $ASK_{a_{i}}=\left\{t_{a_{i}},{\left\{q_{a_{i},k}\right\}}_{1\leq k\leq |S_{a_{i}}|}\right\}$.

#### Key generation

The key generation phase is divided into three parts: signature key generation, attribute private key generation, and user private key generation.

1. Signature key generation
  The Environmental Protection Agency generates signature keys for the monitoring equipment. The EPA makes *r* ∈ *Z*_*p*_ be a random number and calculates *P*_0_ = *MK* ⋅ *g*, *W*_*id*_ = *H*_1_(*id*, *rg*), *Q* = *r* + *W*_*id*_ ⋅ *MK*, then the partial private key of the monitoring equipment is (*Q*, *rg*). The secret value of the monitoring equipment *μ* ∈ *Z*_*p*_ is picked by EPA. The EPA calculates *l* = *μg*, then sets the signature public key as *SPK*_*id*_ = (*l*, *rg*) and the signature private key as *SSK*_*id*_ = (*μ*, *Q*).
2. Attribute private key generation
  The AA generates the attribute private key for the monitoring user from the user’s attribute set. AA selects the attribute private key generation function *AAKeyGen*, which takes the public parameter *Params*, the private key $ASK_{a_{i}}$ of the attribute authority *a*_*i*_, and the attribute set *S*_*id*_ of the monitoring user *id* as input. It calculates $D{}_{a_{i},k}^{\prime }=g^{\alpha {q_{a_{i},k}}^{-1}\cdot {t_{a_{i}}}^{-1}}$ for each attribute $s_{a_{i},k}\in \left(S_{a_{i}}\cap S_{id}\right)$, and output the attribute private key $SK_{a_{i},id}=\left\{D{}_{a_{i},k}^{\prime }\right\}$ of the monitoring user *id*. The attribute authority *a*_*i*_ transmits $SK_{a_{i},id}$ to the EPA to generate the user’s private key.
3. User private key generation
  Environmental Protection Agency generates user private keys for monitoring data users. The EPA selects user private key generation function *EPAKeyGen*, which inputs *MK* and $SK_{a_{i},id}$, let *γ*, *θ* ∈ *Z*_*p*_ be random number for the monitoring data user *id*, and calculates Eq (1).
  $$\begin{array}{ccc} SK_{id} & = & \left\{\begin{array}{c} D=g^{\alpha (\beta +\gamma )},D_{1}=g^{\alpha \gamma }h^{\theta },D_{2}=g^{\theta },\\ D_{a_{i},k}={\left(D{}_{a_{i},k}^{\prime }\right)}^{\gamma }=g^{\alpha \gamma \cdot {q_{a_{i},k}}^{-1}{t_{a_{i}}}^{-1}} \end{array}\right\} \end{array}$$ (1)
  Then the function *EPAKeyGen* outputs the private key $SK_{id}=\left\{D,D_{1},D_{2},{\left\{D_{a_{i},k}\right\}}_{s_{a_{i},k}\in S_{id}}\right\}$ of the environmental monitoring user and sends *SK*_*id*_ to the monitoring user *id*.

#### Monitoring data storage

The monitoring data storage phase is divided into two parts: fog node encryption and monitoring equipment encryption.

1. Fog node encryption
  Data encryption is required before it can be uploaded to the ECS for storage. To save computational overhead, the monitoring equipment transmits access policy to the fog node at first, and the FN encrypts it to obtain partial ciphertext. The monitoring user defines *T*_*a*_ and transmits it to the FN. For each node *x* in the access policy *T*_*a*_, the threshold value *x*_*n*_ of *x* is set to the highest degree of the ratio polynomial *P*_*x*_, that is *f*_*x*_ + 1, the relationship between the two is *x*_*n*_ = *f*_*x*_ + 1. For the root *R*, the fog computing node selects a random number *v* ∈ *Z*_*p*_ to make the root node polynomial as *P_R_*(0) = *v* and the polynomial of each other node *x* as *P*_*x*_(0) = *P*_*parent*(*x*)_(*index*(*x*)), where *parent*(*x*) is the parent node of *x*, the unique index value of each node *x* is *index*(*x*). Take $S_{T_{a}}$ as the collection of leaf nodes in *T*_*a*_, and the fog node computes the partial ciphertext *CT*′ as Eq (2).
  $$\begin{array}{c} CT^{\prime }=\{T_{a},C_{1}=g^{v},C_{2}=h^{v},{\left\{C_{a_{i},k}=g^{q_{a_{i},k}\cdot t_{a_{i}}P_{s_{T_{a}}\left(s_{a_{i},k}\right)}\left(0\right)}\right\}}_{s_{T_{a}}\in S_{T_{a}}}\} \end{array}$$ (2)
2. Monitoring equipment encryption
  After the environmental monitoring equipment re-encrypts the partial ciphertext *CT*′ and plaintext data to obtain the complete ciphertext, the environmental monitoring equipment *id* transfers the ciphertext to the ECS for storage. First of all, the environmental monitoring equipment *id* partially signs the plaintext *M*, calculates *σ*_1_ = *H*_2_(*id*, *l*, *rg*, *P*_0_), *σ*_2_ = *H*_3_(*M*, *id*, *l*, *rg*, *P*_0_) and makes *σ*′ = (*σ*_1_ ⋅ *μ* + *Q*)*σ*_2_, where *σ*′ is the partial signature of the monitoring equipment *id* on the information *M*. Then the monitoring equipment selects random number *w*, *DK* ∈ *Z*_*p*_, makes *DK* as a symmetric key, uses it to encrypt the data plaintext *M* obtains *C* = *SE*_*DK*_(*M*) and embeds the signature *σ*′ in it. The EME calculates *C*_3_ = *DK* ⋅ *e*(*g*, *g*)^*αβw*^, *C*′ = *g*^*w*^, *C*_1_′ = *g^v^* · *g^w^* and *C*_2_′ = *h^v^* · *h^w^*, then generates ciphertext as Eq (3).
  $$\begin{array}{c} CT=\{T_{a},C,C_{3},C^{\prime },{C_{1}}^{\prime },{C_{2}}^{\prime },{\left\{C_{a_{i},k}\right\}}_{s_{T_{a}}\in S_{T_{a}}}\} \end{array}$$ (3)

#### Monitoring data sharing

The monitoring data sharing phase is divided into two parts: fog node decryption and environmental monitoring user decryption.

1. Fog node decryption
  The user of environmental monitoring requests to access data to the environmental cloud server. When the MU’s attributes meet *T*_*a*_, the ECS sends ciphertext to the fog node for partial decryption to obtain partial ciphertext. The FN undertakes most calculations of partial decryption, to save the computing overhead of the monitoring users. The FN calculates *σ*_1_ = *H*_2_(*id*, *l*, *rg*, *P*_0_) and then verifies whether the equation *e*(*σ*′, *g*) = *e*(*σ*_2_, (*σ*_1_ ⋅ *l* + *rg* + *W*_*id*_ ⋅ *P*_0_)) holds. If it is not true, the signature *σ*′ is invalid, the fog node decryption process should be terminated and warns of illegal behavior; if it is true, the signature *σ*′ is valid, and the fog computing node can continue to perform partial decryption. Then the MU obtains partial user’s private key $S{K_{id}}^{\prime }=\left\{D_{1},D_{2},{\left\{D_{a_{i},k}\right\}}_{s_{a_{i},k}\in S_{id}}\right\}$ from the EPA, and the fog node calculates $FogDecryptNode\left(CT,x,SK{}_{id}^{\prime }\right)\to F_{x}$ through the ciphertext *CT* and partial private key *SK_id_*′, where the node *x* is in the *T*_*a*_.
  If the node *x* is a leaf node, when the attribute represented by *x* is included in the MU’s attribute set, the fog node calculates *F*_*x*_ as Eq (4), otherwise $DecryptNode(CT,x,SK{}_{id}^{\prime })=⊥$.
  $$\begin{array}{c} \begin{array}{cc} F_{x} & =e(D_{a_{i},k},C_{a_{i},k})\\ & =e(g^{\alpha \gamma \cdot {q_{a_{i},k}}^{-1},{t_{a_{i}}}^{-1}},g^{q_{a_{i},k}\cdot t_{a_{i}}\cdot P_{s_{T_{a}}\left(s_{a_{i},k}\right)}\left(0\right)})\\ & =e{\left(g,g\right)}^{\alpha \gamma \cdot P_{s_{T_{a}}(s_{a_{i},k)}}\left(0\right)} \end{array} \end{array}$$ (4)
  If the node *x* is non-leaf node, fog node calculates *F_x_*′ = *FogDecryptNode*(*CT*, *x*′, *SK_id_*′) and calculates *F*_*x*_ as Eq (5).
  $$\begin{array}{c} \begin{array}{cc} F_{x} & =\prod_{x^{\prime }\in S_{x}}{F_{x^{\prime }}}^{\Delta index\left(x^{\prime }\right),{S_{x}}^{\prime }\left(0\right)}\\ & =\prod_{x^{\prime }\in S_{x}}{\left(e{\left(g,g\right)}^{\alpha \gamma \cdot P_{parent(x^{\prime })}\left(index\left(x^{\prime }\right)\right)}\right)}^{\Delta index\left(x^{\prime }\right),{S_{x}}^{\prime }\left(0\right)}\\ & =\prod_{x^{\prime }\in S_{x}}{\left(e{\left(g,g\right)}^{\alpha \gamma \cdot P_{x}\left(index\left(x^{\prime }\right)\right)}\right)}^{\Delta index\left(x^{\prime }\right),{S_{x}}^{\prime }\left(0\right)}\\ & =e{\left(g,g\right)}^{\alpha \gamma \cdot P_{x}\left(0\right)} \end{array} \end{array}$$ (5)
  When MU’s attribute set meets *T*_*a*_, the FN calculates the root node *F*_*R*_ as Eq (6), wherein the fog node encryption process, there is a root node polynomial *P*_*R*_(0) = *v*.
  $$\begin{array}{c} \begin{array}{cc} F_{R} & =FogDecryptNode(CT,R,S{K_{id}}^{\prime })\\ & =e{\left(g,g\right)}^{\alpha \gamma \cdot P_{R}\left(0\right)}\\ & =e{\left(g,g\right)}^{\alpha \gamma \cdot v} \end{array} \end{array}$$ (6)
  Then, the fog node decrypts *C*_1_′, *C*_2_′ as the following Eq (7).
  $$\begin{array}{c} \begin{array}{cc} A & =\frac{e(D_{1},{C_{1}}^{\prime })}{e\left(D_{2},{C_{2}}^{\prime }\right)F_{R}}=\frac{e(g^{\alpha \gamma }h^{\theta },g^{v}g^{w})}{e\left(g^{\theta },h^{v}h^{w}\right)e{\left(g,g\right)}^{\alpha \gamma \cdot v}}\\ & =\frac{e{\left(g,g\right)}^{\alpha \gamma (v+w)}\cdot e{\left(h,g\right)}^{\theta (v+w)}}{e{\left(g,h\right)}^{\theta (v+w)}e{\left(g,g\right)}^{\alpha \gamma \cdot v}}\\ & =\frac{e{\left(g,g\right)}^{\alpha \gamma (v+w)}}{e{\left(g,g\right)}^{\alpha \gamma \cdot v}}\\ & =e{\left(g,g\right)}^{\alpha \gamma \cdot w} \end{array} \end{array}$$ (7)
  The fog node resigns the partial signature *σ*′ embedded in *C* = *SE*_*DK*_(*M*). The fog node selects a random number *δ* ∈ *Z*_*p*_, and calculates *σ*_1_ = *H*_2_(*id*, *l*, *rg*, *P*_0_), *σ*_1_′ = *σ*_1_ · *δ*, then the fog node re-signs *σ* = (*σ*_1_′ · *μ* + *Q*)*σ*_2_ and re-embeds it in *C*. The partial ciphertext decrypted by the fog node is *CT*′ = {*C*, *C*_3_, *C*′, *A*}.
2. Environmental monitoring user decryption
  After the fog node performs partial decryption, the monitoring data user only needs very few calculations to recover the plaintext. The fog node first sends the partial ciphertext *CT*′ = {*C*, *C*_3_, *C*′, *A*} to the environmental monitoring user. After the MU receives partial ciphertext *CT*′ from the fog node, the validity of the signature *σ* is also verified. The monitoring user calculates *σ*_1_ = *H*_2_(*id*, *l*, *rg*, *P*_0_), *σ*_1_′ = *σ*_1_ · *δ*, and then verifies whether the equation *e*(*σ*, *g*) = *e*(*σ*_2_, (*σ*_1_′ · *l* + *rg* + *W_id_* · *P*_0_)) holds. If it is not established, the signature is invalid, and the user’s decryption process should be terminated and warns of illegal behavior; if it is established, the signature is valid, and the monitoring user can continue the decryption operation. The MU uses *SK*_*id*_ to perform decryption operations and acquires the symmetric key *DK*, the calculation process as Eq (8).
  $$\begin{array}{c} \begin{array}{cc} DK & =\frac{C_{3},A}{e(D,C^{\prime })}=\frac{DK\cdot e{\left(g,g\right)}^{\alpha \beta w}\cdot e{\left(g,g\right)}^{\alpha \gamma w}}{e(g^{\alpha (\beta +\gamma )},g^{w})}\\ & =\frac{DK\cdot e{\left(g,g\right)}^{\alpha \beta w}\cdot e{\left(g,g\right)}^{\alpha \gamma w}}{e\left(g^{\alpha \beta },g^{w}\right)\cdot e\left(g^{\alpha \gamma },g^{w}\right)}\\ & =\frac{DK\cdot e{\left(g,g\right)}^{\alpha \beta w}\cdot e{\left(g,g\right)}^{\alpha \gamma w}}{e{\left(g,g\right)}^{\alpha \beta w}\cdot e{\left(g,g\right)}^{\alpha \gamma w}}=DK \end{array} \end{array}$$ (8)
  Finally, the environmental monitoring user uses the symmetric key *DK* decrypt *C* = *SE*_*DK*_(*M*) to obtain the plaintext.

#### Revocation

This phase mainly solves the problem of user or attribute permission changes that may occur, and includes the following four steps.

1. Revocation of monitoring equipment and users
  When any monitoring device is attacked and the wrong data is transmitted, the signature verification becomes invalid. Or if any data user is an illegal user and attempts to obtain plaintext messages through illegal channels, the equipment or monitoring user shall be integrated revoke from the system. The environmental cloud server deletes the unique identity value *id* of the monitoring equipment or monitoring user from the equipment and user list *U*. Any monitoring equipment can send monitoring data to the nearby FN, but the FN receives data only if the signature of the data from the EME in the list *U* is verified. In the same way, any monitoring user could download *CT* on the ECS, while only legitimate users in the list *U* can obtain the key to decrypt *CT*, which ensures the security of the system.
2. Revocation of attribute
  The attribute authority selects a random number *τ* and assigns it to EPA, ECS and related users who need revoke attributes, makes *τ* as a re-encryption parameter. The attribute authority updates the attributes which it manages, and the attribute private key is updated to $SK_{a_{i},id}=\left\{{D_{a_{i},k}}^{\prime }=g^{\alpha {q_{a_{i},k}}^{-1}\cdot {t_{a_{i}}}^{-1}+\tau }\right\}$.
3. Update of user key
  AA updates the attribute private key and send it to the EPA. EPA selects the user’s private key generation function to obtain a new key. The updated user private key as $SK_{id}=\left\{D,D_{1},D_{2},{\left\{D_{a_{i},k}=g^{\alpha \gamma \cdot {q_{a_{i},k}}^{-1}\cdot {t_{a_{i}}}^{-1}+\tau \gamma }\right\}}_{s_{a_{i},k}\in S_{id}}\right\}$.
4. Update of ciphertext
  The environmental cloud server updated the ciphertext as the following Eq (9).
  $$\begin{array}{c} CT^{*}=\{\begin{array}{c} T_{a},C,C_{3}=DK\cdot e{\left(g,g\right)}^{\alpha \beta w+\tau },C^{\prime }=g^{w+\tau }{C_{1}}^{\prime }=g^{(v+w)+\tau },\\ {C_{2}}^{\prime }=h^{(v+w)+\tau }{\left\{C_{a_{i},k}=g^{q_{a_{i},k}\cdot t_{a_{i}}\cdot P_{s_{T_{a}}(s_{a_{i},k)}}\left(0\right)+\tau }\right\}}_{s_{T_{a}}\in S_{T_{a}}} \end{array}\} \end{array}$$ (9)

## Security analysis

### Signature correctness

After the monitoring equipment *id* partially signs the plaintext *M*, the signature verification process is as Eq (10).

$$\begin{array}{c} \begin{array}{cc} e(\sigma^{\prime },g) & =e\left(\sigma_{2},\left(\sigma_{1}\cdot l+rg+W_{id}\cdot P_{0}\right)\right)=e\left(\left(\sigma_{1}\cdot \mu +Q\right)\sigma_{2},g\right)\\ & =e(\left(\sigma_{1}\cdot \mu +r+W_{id}\cdot MK\right)\cdot g,\sigma_{2})\\ & =e(\sigma_{2},\left(\sigma_{1}\cdot \mu g+rg+W_{id}\cdot MK\cdot g\right)) \end{array} \end{array}$$ (10)

In the same way, the fog computing node’s re-signature verification process for partial signatures is as Eq (11).

$$\begin{array}{c} \begin{array}{c} e\left(\sigma ,g\right)=e\left(\sigma_{2},\left({\sigma_{1}}^{\prime }\cdot l+rg+W_{id}\cdot P_{0}\right)\right)=e\left({\sigma_{1}}^{\prime }\cdot \mu +Q\right)\cdot \sigma_{2},g)\\ =e\left(\sigma_{1}\cdot \delta \mu +r+W_{id}\cdot MK\right)\cdot g,\sigma_{2})\\ =e(\sigma_{2},\left(\sigma_{1}\cdot \delta \mu g+rg+W_{id}\cdot MK\cdot g\right)) \end{array} \end{array}$$ (11)

If the equation does not hold, the verification fails and the signature of the data information is invalid.

### Data confidentiality

First, the access policy is used to encrypt the environmental monitoring data. If the monitoring user does not have a valid attribute set that meets the access policy, the access will be denied to ensure data confidentiality. During the encryption phase, although the FN performs partial encryption calculations for environmental monitoring equipment, it is still unable to access the data without a private key. In the decryption phase, illegal monitoring users cannot acquire the correct user private key because the attribute set does not meet the access policy in ciphertext. Neither the environmental cloud server nor the fog computing node can compute to restore the value *A* = *e*(*g*, *g*)^*αγ*⋅*w*^ and acquire the required symmetric key *DK*. Therefore, only legitimate monitoring users who have valid attributes and meet the access policy could decrypt the ciphertext. (see S1 Appendix for the concrete proof process).

### Data unforgeability

Digital signatures can achieve data unforgeability very well. Without obtaining the unique identity value *id* of the terminal device and the signature private key generated by it, no one can forge a valid signature. In the environmental monitoring application scenario, if the monitoring collection equipment is compromised, it wants to upload false plaintext information after tampering. However, malicious attackers cannot know the signature private key *SSK*_*id*_ through public parameter, so a valid signature cannot be forged. An invalid signature cannot be verified by the ECS. If the signature is invalid, the message is not true and the ECS will not store the corresponding data. (See S2 Appendix for the concrete proof process).

### Distributed multi-authorization

There are serious security risks in the mode of single-authorized organization. Once the only authorized authority is controlled by an illegal attacker, or the key spamming occurs due to excessive authority, it may obscurely distribute private keys beyond their access rights to some illegal users. This causes the entire system not to crash immediately, but illegal users can continuously obtain arbitrary data files, which bring serious security threats. Our proposal uses a multi-authorization method to establish distributed nodes. As the central authority, EPA only takes part in the system initialization phase, and does not take part in the management of user attributes and keys. This avoids the security problems caused by the excessive authority of the central authority EPA, which forms a trusted distributed system.

### Collusion resistance

The authorized agency EPA generates private keys for different monitoring users in our scheme. The private keys are related to random numbers *γ*, *θ*. The random numbers generated for each user are different and have a unique correlation with each user, which makes the new private key formed by combining parts of different private keys with each other meaningless. Even if there is a situation where the combination of different attribute sets of multiple users can meet the access policy, they are unable to calculate *F*_*R*_ = *e*(*g*, *g*)^*αγ*⋅*v*^ in the outsourcing decryption phase because they could not obtain the correct user private key. In summary, our proposal is resistant to collusion.

### Privacy protection

The system assigns the attribute set managed by each attribute authority AA according to the complete set of attributes. Attributes of the same type can be managed by the same attribute authority. Because different attribute authorities manage different attribute sets, it is impossible to know the specific attribute of the user, which protects the privacy of the user to a certain extent. Multiple different authorities exist and operate independently, without even knowing the existence of other attribute authorities. Such a distributed system can also effectively prevent collusion at the same time.

## Performance analysis

This section mainly compares the performance of our proposal with several existing data sharing schemes. The performance analysis of our proposal is mainly evaluated from two aspects: communication overhead and computation overhead. The definition of notations in the analysis is shown in Table 1.

**Table 1:** Notations adopted in our paper.

Symbols	Representations
${\bar{G}}_{0}$	Length of each element in the group *G*_0_
${\bar{G}}_{T}$	Length of each element in the group *G*_*T*_
*n*	Total number of attributes in the system
*k*	Number of attributes in the data Visitor *id* attribute set
*c*	Number of attributes used for encryption
*d*	Number of attributes used for decryption
$\bar{H}$	Length of access policy
$E_{G_{0}}$	An exponential operation on the group *G*_0_
$E_{G_{T}}$	An exponential operation on the group *G*_*T*_
*P*	A bilinear pairing operation

The scheme in paper [6] does not have functions such as fog computing outsourcing capabilities and multiple authorizations. Scheme [19] realizes encryption and decryption outsourcing computing in a fog environment, but it is only suitable for a single authorized institution. Scheme [20] realizes multi-authorization agency attribute-based encryption with revocation function, but it is not suitable for fog environments and does not implement outsourcing of encryption and decryption calculations. Scheme [18] implements encryption and decryption calculation outsourcing under the cloud architecture, but does not implement the revocation function. Our scheme achieves multi-authorization attribute base encryption and decryption with outsourced computing and revocable capabilities under the cloud-fog computing. Signatures are introduced on the terminal device side to achieve message verifiability. And outsource partial encryption and decryption operations to the fog node, which reduces burden on the data access users and the monitoring equipment. As shown in Table 2, we compare our proposal with other advanced schemes. The result shows that our proposal supports abundant functions.

**Table 2:** The functions comparison.

Schemes	Cloud-fog architecture	Outsourcing computing	Multi-authority	Revocation	Verifiability
[6]	×	×	×	×	×
[18]	√	√	×	×	×
[19]	√	√	×	×	×
[20]	×	×	√	√	×
Ours	√	√	√	√	√

×: not supported; √: supported.

Table 3 shows the communication overhead comparisons with related schemes. We mainly consider the cost caused by the transmission of messages in the communication process, including the length of public key, the length of private key, and the ciphertext size. They are the main measure of communication costs. The public key length of schemes scheme [19, 20] is affected by the number of attributes. If the number of system attributes is large, the communication cost of our proposal is significantly lower than the above two schemes. Although the public key length of our proposal is longer than scheme [18], the private key length and the size of ciphertext are both smaller than the scheme [18], so the total communication overhead is lower than the scheme [18].

**Table 3:** Comparison of communication cost.

Schemes	Public Key Size	Secret Key Size	Ciphertext Size
[18]	$3{\bar{G}}_{0}+{\bar{G}}_{T}$	$\left(4+k\right){\bar{G}}_{0}$	$(4+c){\bar{G}}_{0}+{\bar{G}}_{T}+\bar{H}$
[19]	$(3+n){\bar{G}}_{0}+{\bar{G}}_{T}$	$\left(3+k\right){\bar{G}}_{0}$	$(3+c){\bar{G}}_{0}+{\bar{G}}_{T}+\bar{H}$
[20]	$n{\bar{G}}_{0}+n{\bar{G}}_{T}$	$2k{\bar{G}}_{0}$	$(1+2c){\bar{G}}_{0}+\left(1+c\right){\bar{G}}_{T}+\bar{H}$
Ours	$4{\bar{G}}_{0}+{\bar{G}}_{T}$	$\left(3+k\right){\bar{G}}_{0}$	$(3+c){\bar{G}}_{0}+{\bar{G}}_{T}+\bar{H}$

We also compared the computational cost of our scheme and related schemes, mainly including the computation overhead of encryption operations and decryption operations. The running time of our scheme is mainly distributed in exponential operation and bilinear pairing operation, and the multiplication calculation is negligible. Table 4 shows the computational cost comparison with related schemes, where E represents an exponential operation, and P represents a bilinear pairing operation. Our proposal uses outsourcing encryption and decryption technology to make the fog node bear more calculation pressure. Therefore, users only need to perform a few calculations to complete the decryption. Without the support of outsourcing computing, users will undertake huge computing costs.

**Table 4:** Comparison of computation cost.

Schemes	Encryption	Decryption
[18]	$\left(4+2c\right)E_{G_{0}}+E_{G_{T}}$	$dE_{G_{T}}+\left(4+d\right)P$
[19]	$\left(4+c\right)E_{G_{0}}+2E_{G_{T}}$	$dE_{G_{T}}+4P$
[20]	$\left(1+4c\right)E_{G_{0}}+\left(1+c\right)E_{G_{T}}$	$dE_{G_{T}}+\left(2d+1\right)P$
Ours	$\left(4+c\right)E_{G_{0}}+E_{G_{T}}$	$dE_{G_{T}}+4P$

**Fig 4:**
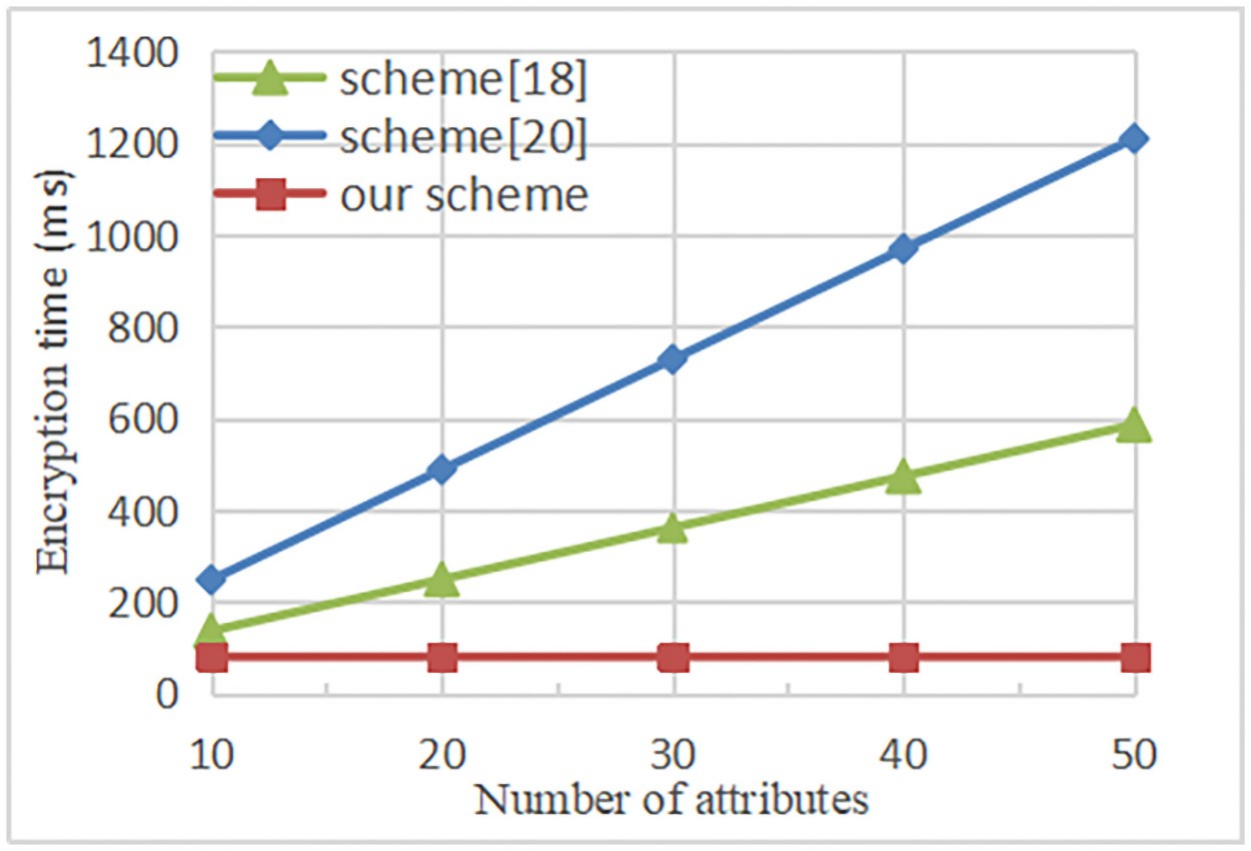
Time cost in encryption.

To compare the computational efficiency, a simulation experiment was carried out. The computer is configured with 4GB RAM, 64-bit Windows 10 operating system and 3GHz Intel Core i5–7400 CPU. The experimental simulation based on Pairing-Based Crypto (PBC) library in the VC++ 6.0 environment. The connection between the time of encryption and the number of attributes is shown in Fig 4. Our proposal outsources partial encryption calculation to the fog node. Therefore, its running time is at a constant level, independent of the attributes number in access policy. The result shows that in the scheme [18, 20], the running time of encryption is linearly related to the attributes number in the access policy.

**Fig 5:**
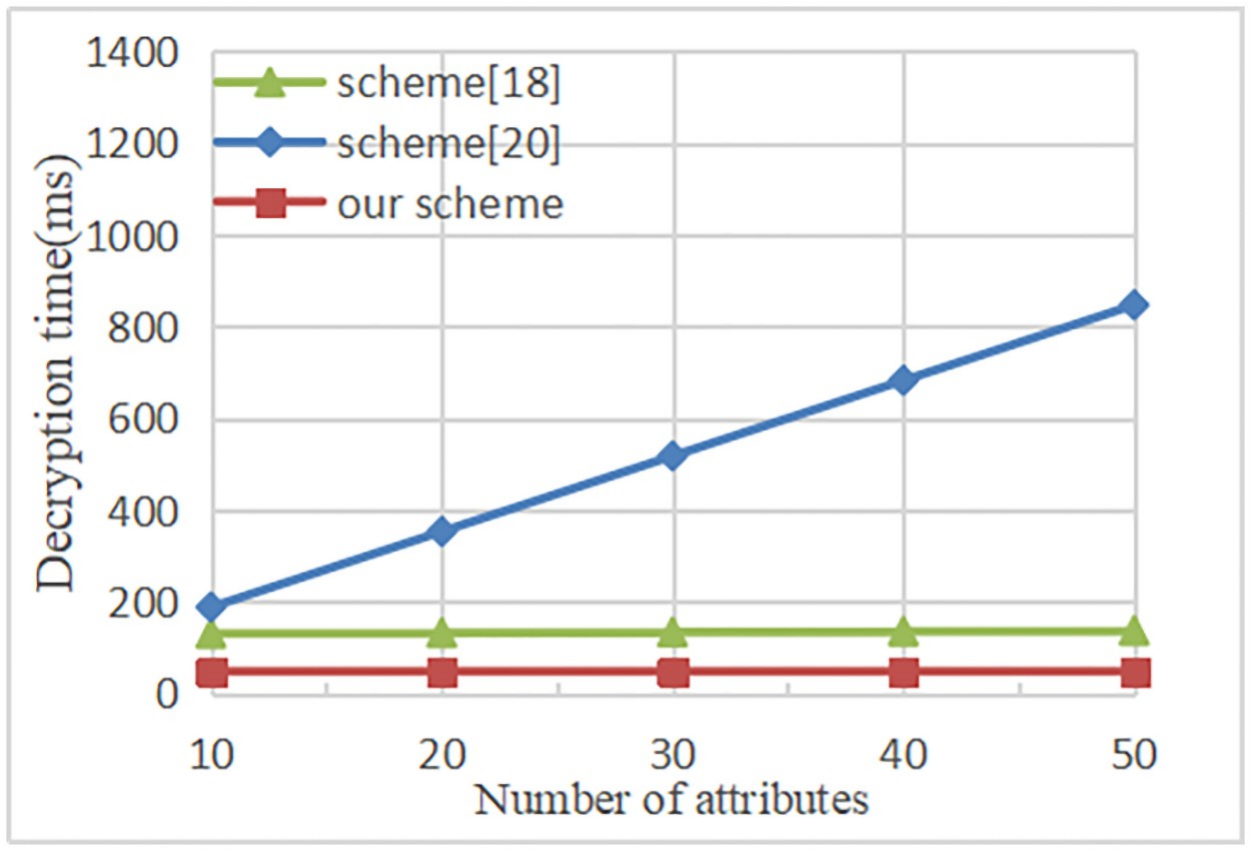
Time cost in decryption.

The relationship between the time of the decryption and the attributes number is shown in Fig 5. Our proposal and scheme [18] outsource partial decryption calculation to the fog node. Therefore, its running time is constant, independent of the attributes number in the access policy. And the decryption time of our scheme is slightly lower than scheme [18]. However, in the literature [20], the running time of decryption is linearly related to the attributes number in the access policy. In general, because there will be lots of data sharing access operations, decryption operations will be more frequent than encryption operations. As the figure shows that the decryption time is accurate to milliseconds as a unit, which fully reflects the high efficiency of the scheme. The experimental result shows that our proposal outsources partial encryption and decryption operations to the fog node, the overall computational cost is lower than the scheme [18, 20].

## Conclusion

With the application of various environmental monitoring technologies, great breakthroughs have been made in the field of environmental protection. However, due to potential public insecure channels environmental monitoring data will still be affected by various insecurities. In our work, we have proposed a secure and efficient environmental monitoring data sharing scheme based on cloud-fog computing, which can achieve the secure collection, storage and sharing of environmental monitoring data. Firstly, our proposal combines cloud-fog computing and attribute-based encryption to realize secure sharing and privacy protection of environmental monitoring data. Secondly, we use digital signatures to realize the integrity, verifiability, and unforgeability of monitoring data. The revocation mechanism is used to improve the flexibility of the system; the distributed multi-authority is used to make the scheme resistant to collusion. Moreover, our proposal also uses outsourcing computing technology. Most of the encryption and decryption computing operations can be executed by fog computing node, which greatly eases the computing burden on devices and users. Furthermore, our scheme has proved its security in the case of Chosen-plaintext Attack and its unforgeability in the case of Chosen Message Attack. The performance analysis shows that our proposal is efficient and has a certain application value in the practical environment of environmental monitoring. For future work, we will implement our proposal on blockchain technology and smart contracts to better realize environmental monitoring data sharing.

## Supporting information

S1 Fig Sensor devices. We cropped and modified the image to the components of Fig 2. Image URL: https://pixabay.com/illustrations/energy-sensor-flow-meter-5444868/ Image by Юрий Коврижных from Pixabay. Pixabay License: Free for commercial use. No attribution required. You can make modifications to content from Pixabay.
(TIF)

S2 Fig Black key. We cropped and modified the image to the components of Fig 2. Image URL: https://pixabay.com/vectors/key-art-vintage-keys-antique-311986/ Image by Clker-Free-Vector-Images from Pixabay. Pixabay License: Free for commercial use. No attribution required. You can make modifications to content from Pixabay.
(TIF)

S3 Fig Yellow key. We cropped and modified the image to the components of Fig 2. Image URL: https://pixabay.com/vectors/brass-gradient-key-1293947/ Image by OpenClipart-Vectors from Pixabay. Pixabay License: Free for commercial use. No attribution required. You can make modifications to content from Pixabay.
(TIF)

S4 Fig Cloud server. We cropped and modified the image to the components of Fig 2. Image URL: https://pixabay.com/illustrations/cloud-computer-circuit-board-cpu-6532831/ Image by akitada31 from Pixabay. Pixabay License: Free for commercial use. No attribution required. You can make modifications to content from Pixabay.
(TIF)

S5 Fig Fog node. We cropped and modified the image to the components of Fig 2. Image URL: https://pixabay.com/vectors/cloud-cloudy-sun-weather-mist-159393/ Image by OpenClipart-Vectors from Pixabay. Pixabay License: Free for commercial use. No attribution required. You can make modifications to content from Pixabay.
(TIF)

S6 Fig Drone. We cropped and modified the image to the components of Fig 2. Image URL: https://pixabay.com/vectors/aerial-air-drone-flight-2024891/ Image by OpenClipart-Vectors from Pixabay. Pixabay License: Free for commercial use. No attribution required. You can make modifications to content from Pixabay.
(TIF)

S7 Fig Monitor. We cropped and modified the image to the components of Fig 2. Image URL: https://pixabay.com/vectors/smart-home-house-technology-2005993/ Image by Pixaline from Pixabay. Pixabay License: Free for commercial use. No attribution required. You can make modifications to content from Pixabay.
(TIF)

S8 Fig Landscape. We cropped and modified the image to the components of Fig 2. Image URL: https://pixabay.com/vectors/pond-garden-crane-japanese-serene-3046592/ Image by Debi Brady from Pixabay. Pixabay License: Free for commercial use. No attribution required. You can make modifications to content from Pixabay.
(TIF)

S9 Fig Environmental protection agency. We cropped and modified the image to the components of Fig 2. Image URL: https://pixabay.com/vectors/building-house-architecture-city-2097690/ Image by robotSchnoubab from Pixabay. Pixabay License: Free for commercial use. No attribution required. You can make modifications to content from Pixabay.
(TIF)

S10 Fig Attribute authority. We cropped and modified the image to the components of Fig 2. Image URL: https://pixabay.com/illustrations/architecture-buildings-skyscrapers-5594350/ Image by andrezin_ce from Pixabay. Pixabay License: Free for commercial use. No attribution required. You can make modifications to content from Pixabay.
(TIF)

S11 Fig Monitoring user. We cropped and modified the image to the components of Fig 2. Image URL: https://pixabay.com/illustrations/woman-business-desk-office-3132627/ Image by Sabrina Young from Pixabay. Pixabay License: Free for commercial use. No attribution required. You can make modifications to content from Pixabay.
(TIF)

S1 Appendix (PDF)

S2 Appendix (PDF)
