# Supplementary figures and images for: An environmental monitoring data sharing scheme based on attribute encryption in cloud-fog computing
Source: PLoS One. 2021 Sep 30;16(9):e0258062. doi: 10.1371/journal.pone.0258062 (PMC8483347; doi:10.1371/journal.pone.0258062)

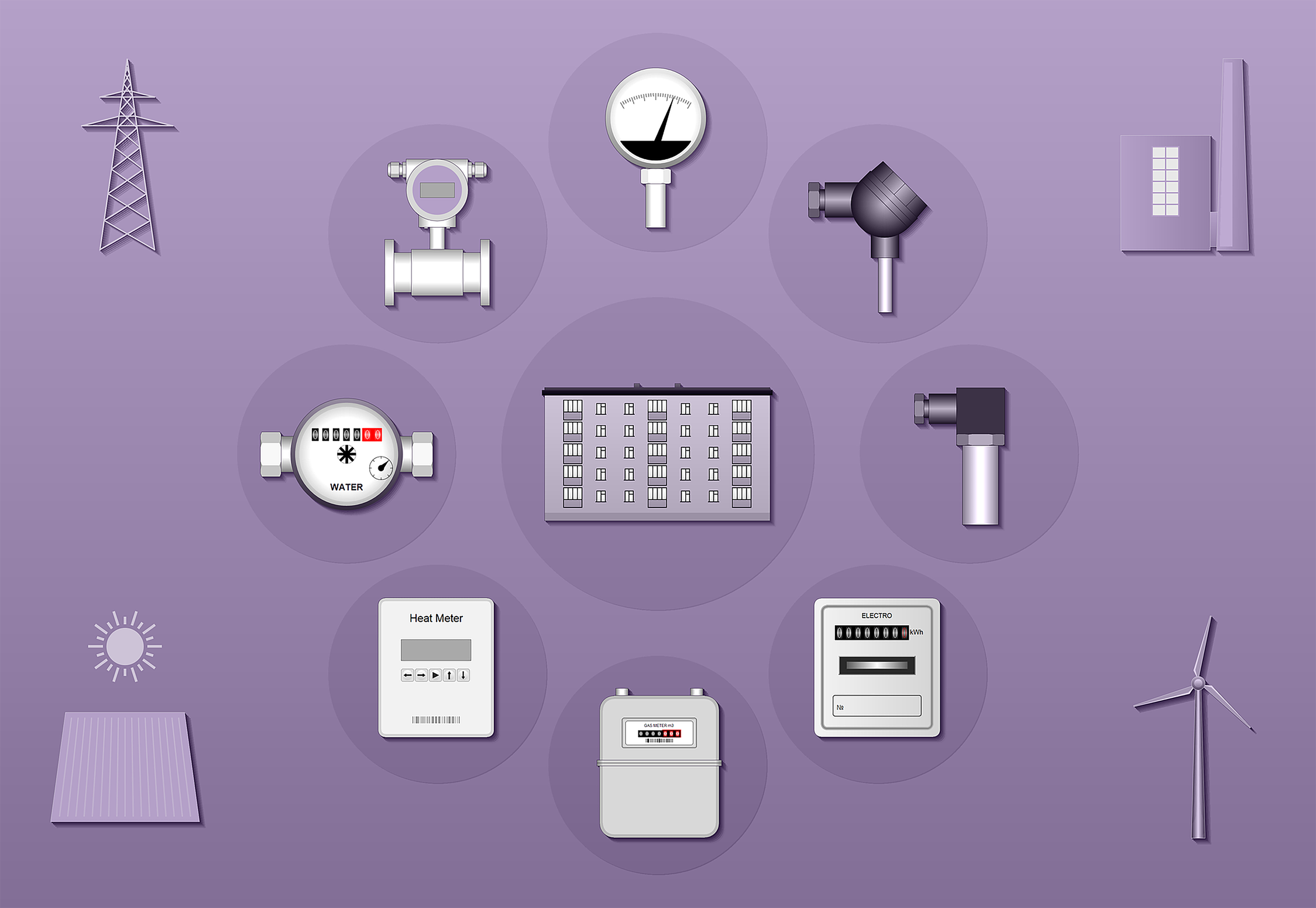

Supplement: S1 Fig — We cropped and modified the image to the components of Fig 2. Image URL: https://pixabay.com/illustrations/energy-sensor-flow-meter-5444868/ Image by Юрий Коврижных from Pixabay. Pixabay License: Free for commercial use. No attribution required. You can make modifications to content from Pixabay. (TIF) [file pone.0258062.s001.tif]

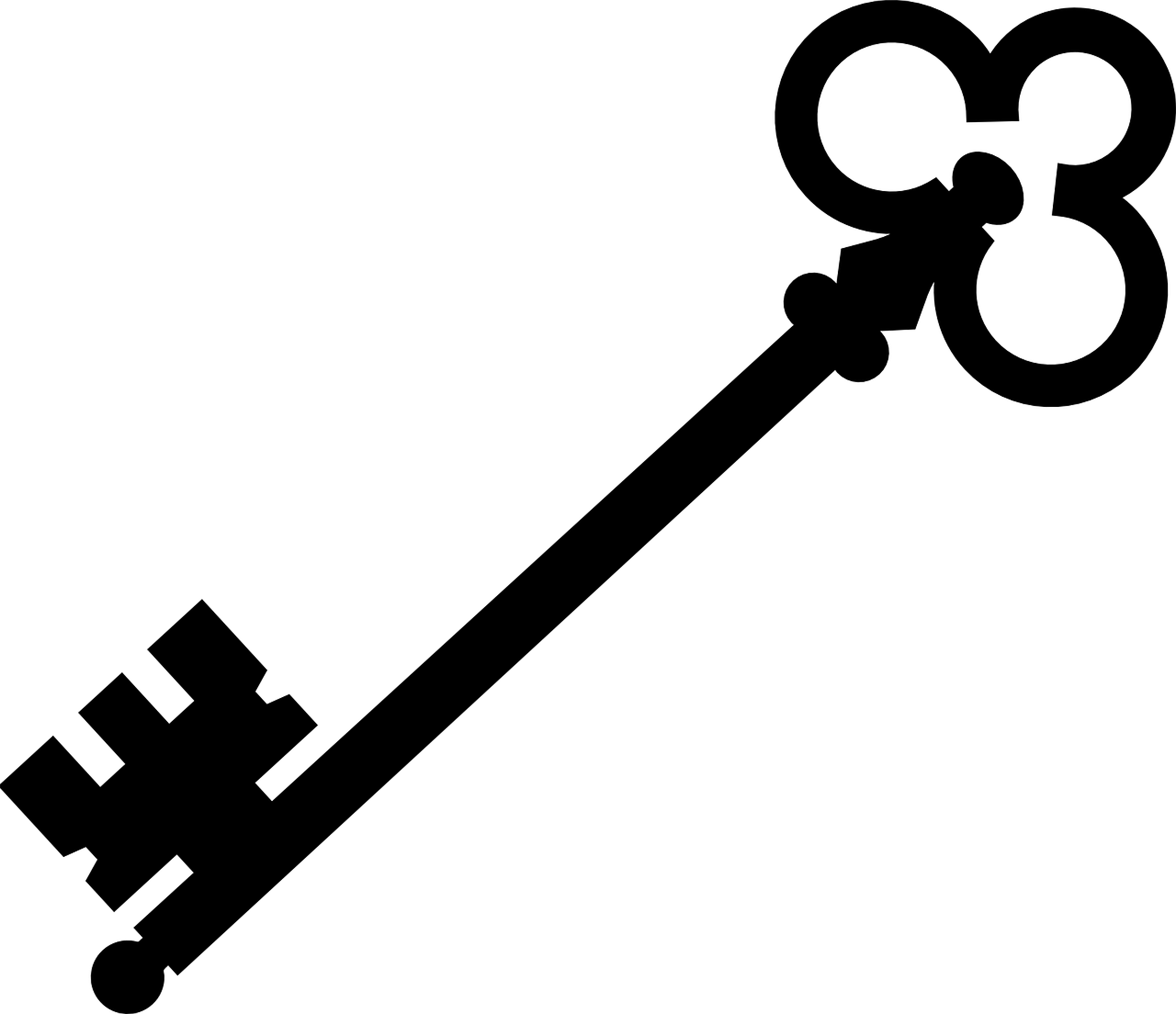

Supplement: S2 Fig — We cropped and modified the image to the components of Fig 2. Image URL: https://pixabay.com/vectors/key-art-vintage-keys-antique-311986/ Image by Clker-Free-Vector-Images from Pixabay. Pixabay License: Free for commercial use. No attribution required. You can make modifications to content from Pixabay. (TIF) [file pone.0258062.s002.tif]

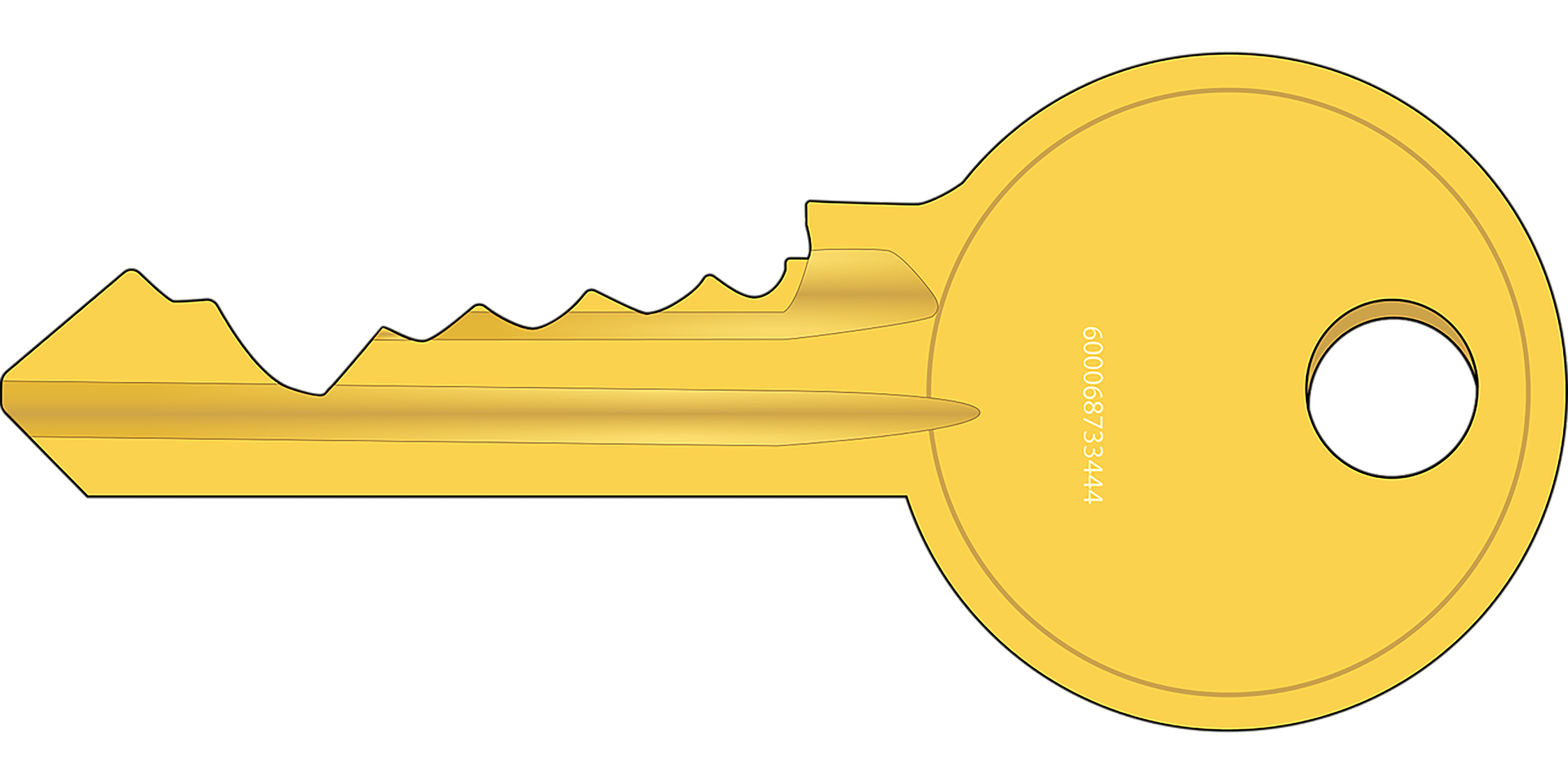

Supplement: S3 Fig — We cropped and modified the image to the components of Fig 2. Image URL: https://pixabay.com/vectors/brass-gradient-key-1293947/ Image by OpenClipart-Vectors from Pixabay. Pixabay License: Free for commercial use. No attribution required. You can make modifications to content from Pixabay. (TIF) [file pone.0258062.s003.tif]

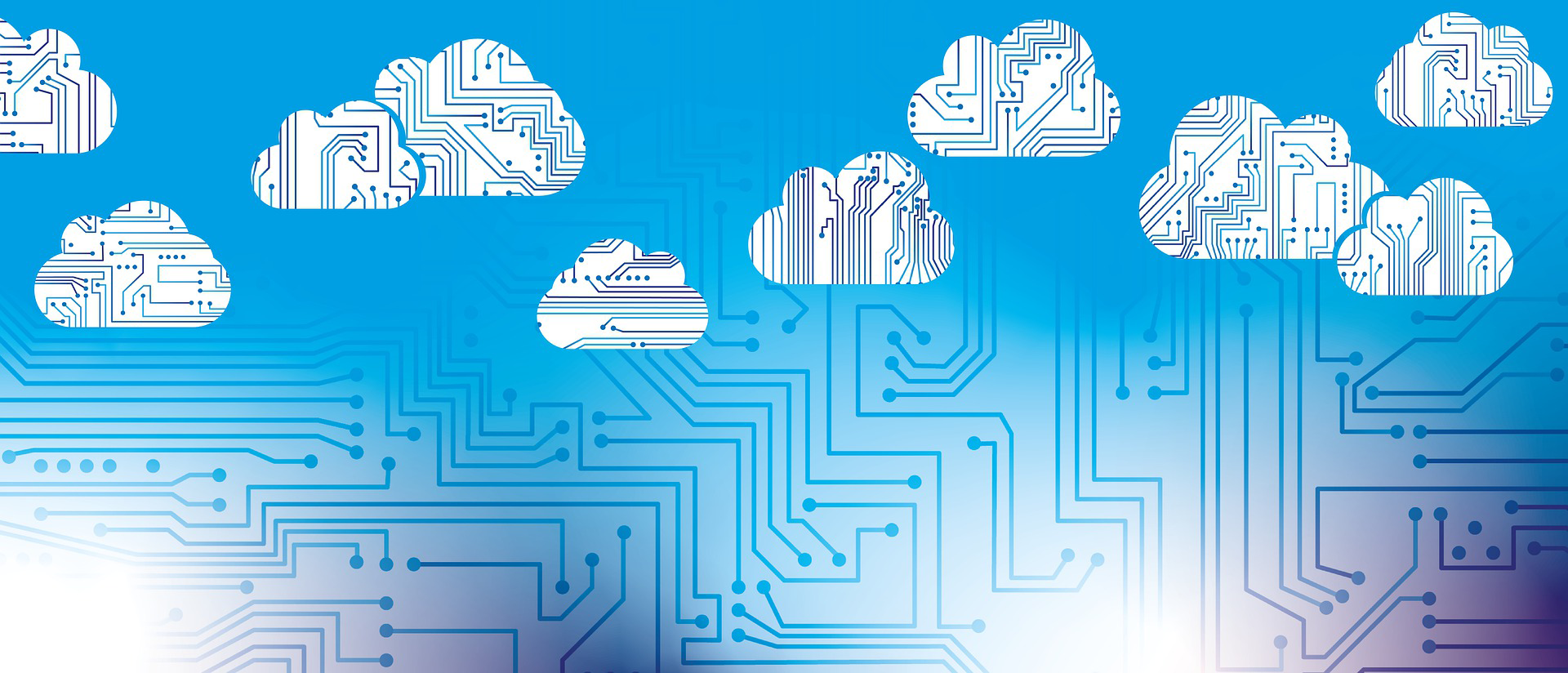

Supplement: S4 Fig — We cropped and modified the image to the components of Fig 2. Image URL: https://pixabay.com/illustrations/cloud-computer-circuit-board-cpu-6532831/ Image by akitada31 from Pixabay. Pixabay License: Free for commercial use. No attribution required. You can make modifications to content from Pixabay. (TIF) [file pone.0258062.s004.tif]

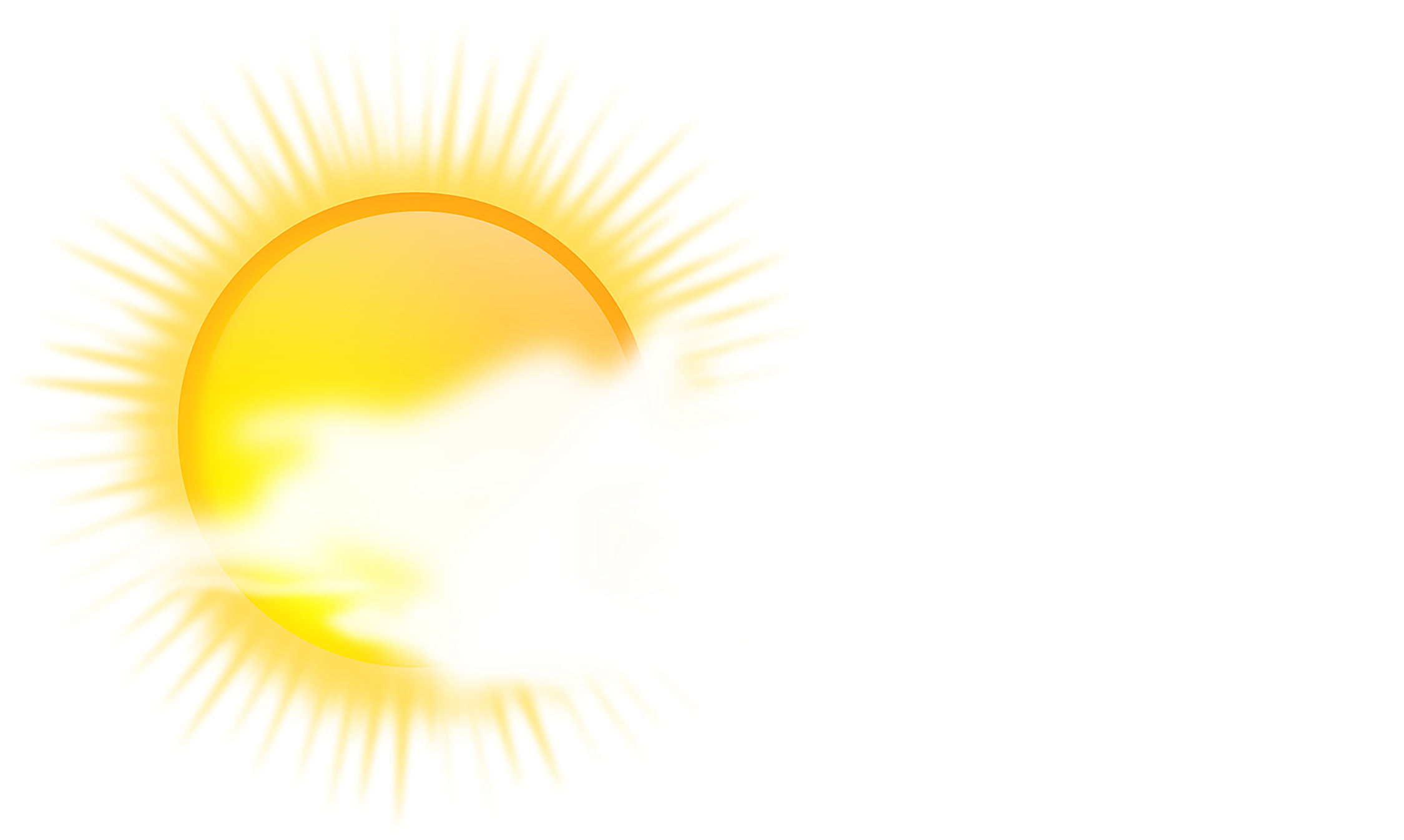

Supplement: S5 Fig — We cropped and modified the image to the components of Fig 2. Image URL: https://pixabay.com/vectors/cloud-cloudy-sun-weather-mist-159393/ Image by OpenClipart-Vectors from Pixabay. Pixabay License: Free for commercial use. No attribution required. You can make modifications to content from Pixabay. (TIF) [file pone.0258062.s005.tif]

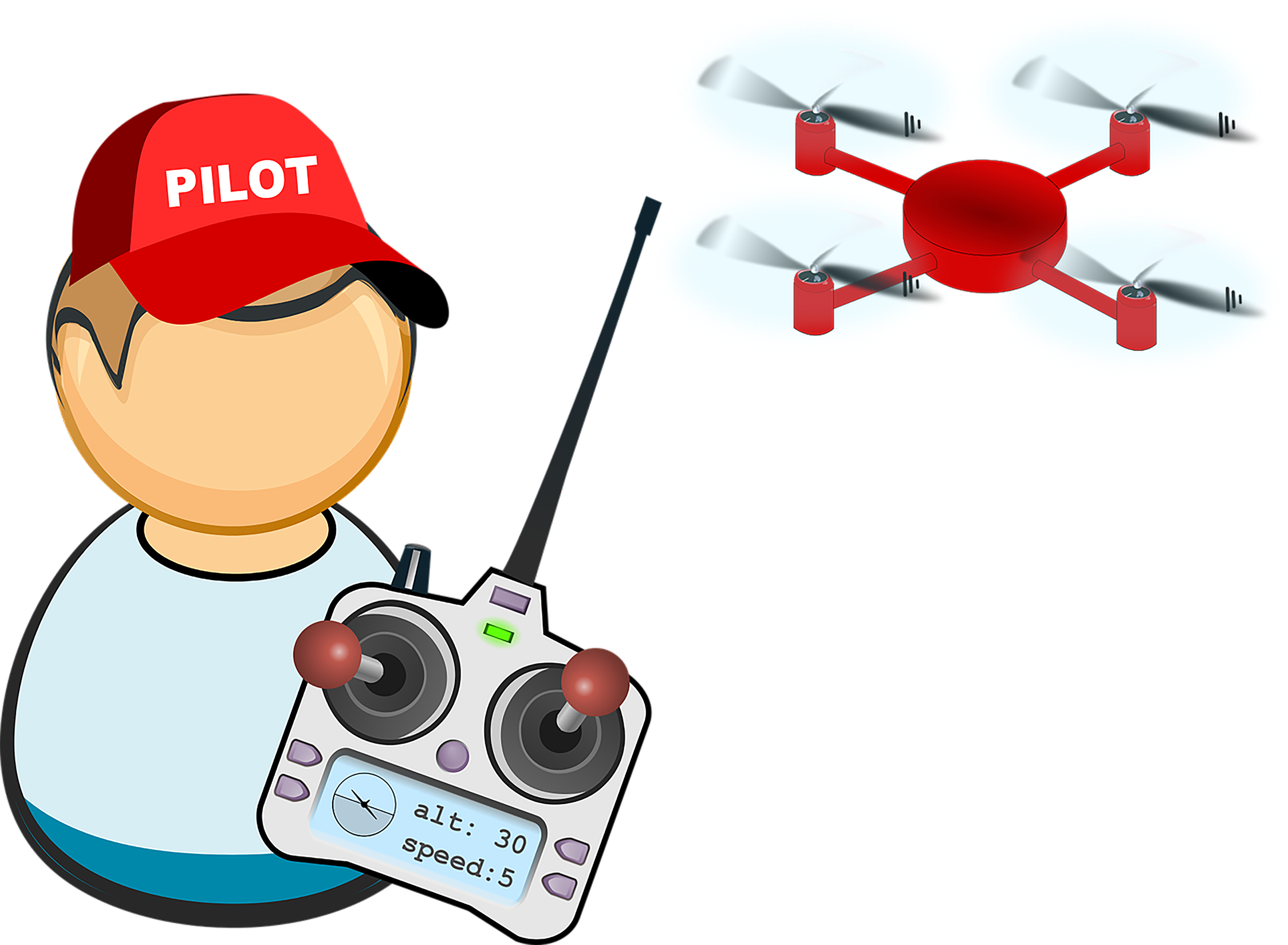

Supplement: S6 Fig — We cropped and modified the image to the components of Fig 2. Image URL: https://pixabay.com/vectors/aerial-air-drone-flight-2024891/ Image by OpenClipart-Vectors from Pixabay. Pixabay License: Free for commercial use. No attribution required. You can make modifications to content from Pixabay. (TIF) [file pone.0258062.s006.tif]

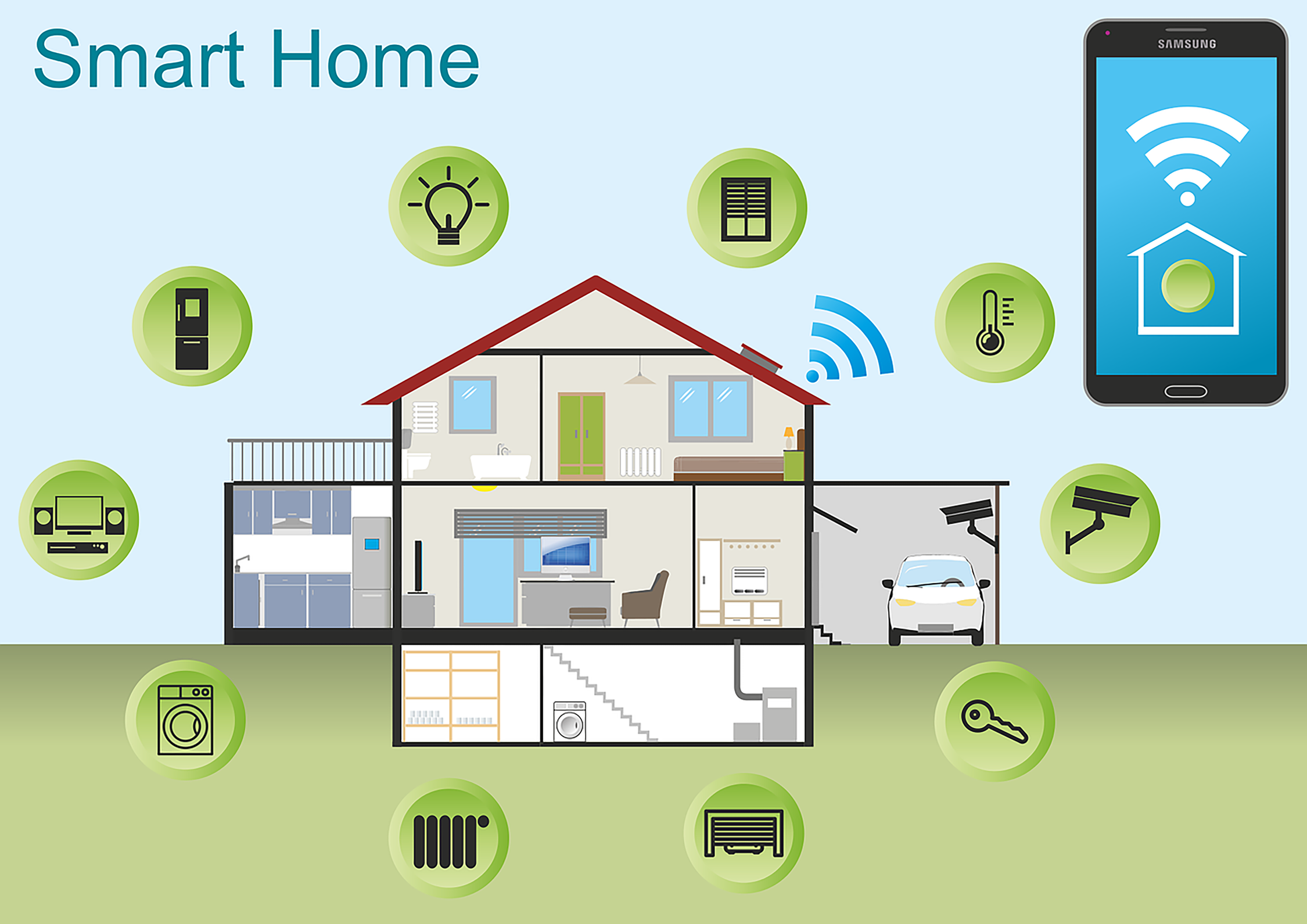

Supplement: S7 Fig — We cropped and modified the image to the components of Fig 2. Image URL: https://pixabay.com/vectors/smart-home-house-technology-2005993/ Image by Pixaline from Pixabay. Pixabay License: Free for commercial use. No attribution required. You can make modifications to content from Pixabay. (TIF) [file pone.0258062.s007.tif]

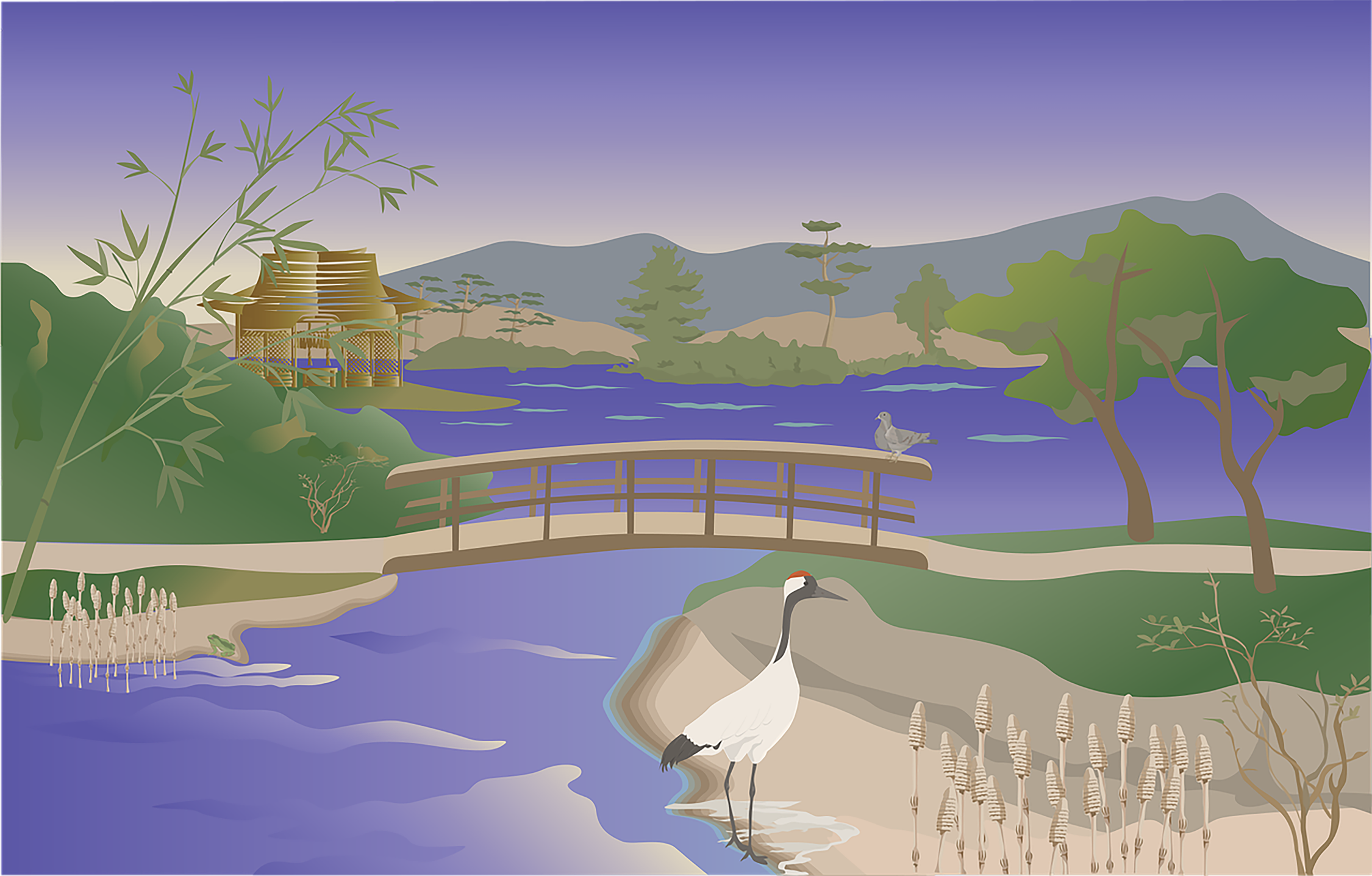

Supplement: S8 Fig — We cropped and modified the image to the components of Fig 2. Image URL: https://pixabay.com/vectors/pond-garden-crane-japanese-serene-3046592/ Image by Debi Brady from Pixabay. Pixabay License: Free for commercial use. No attribution required. You can make modifications to content from Pixabay. (TIF) [file pone.0258062.s008.tif]

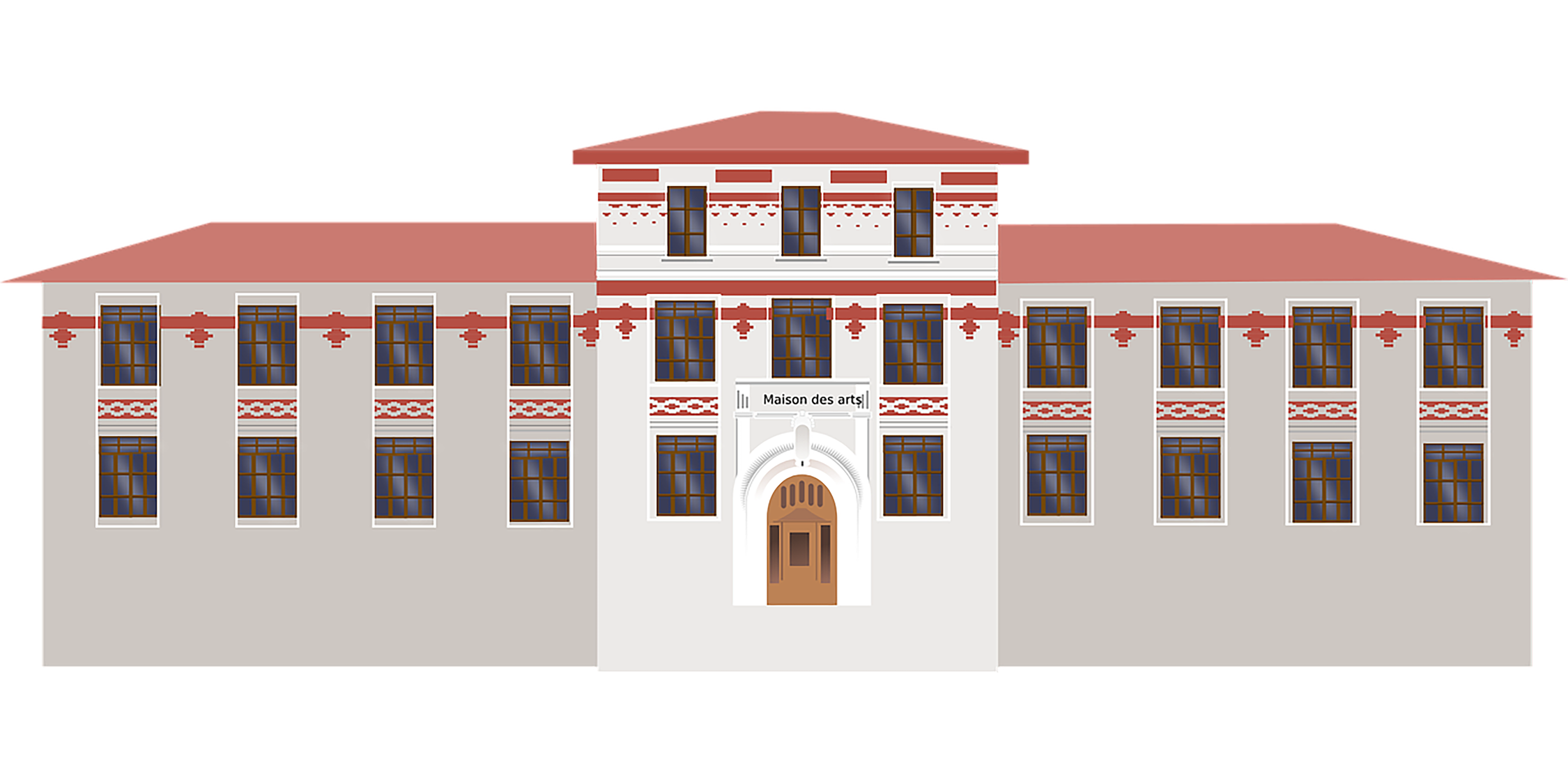

Supplement: S9 Fig — We cropped and modified the image to the components of Fig 2. Image URL: https://pixabay.com/vectors/building-house-architecture-city-2097690/ Image by robotSchnoubab from Pixabay. Pixabay License: Free for commercial use. No attribution required. You can make modifications to content from Pixabay. (TIF) [file pone.0258062.s009.tif]

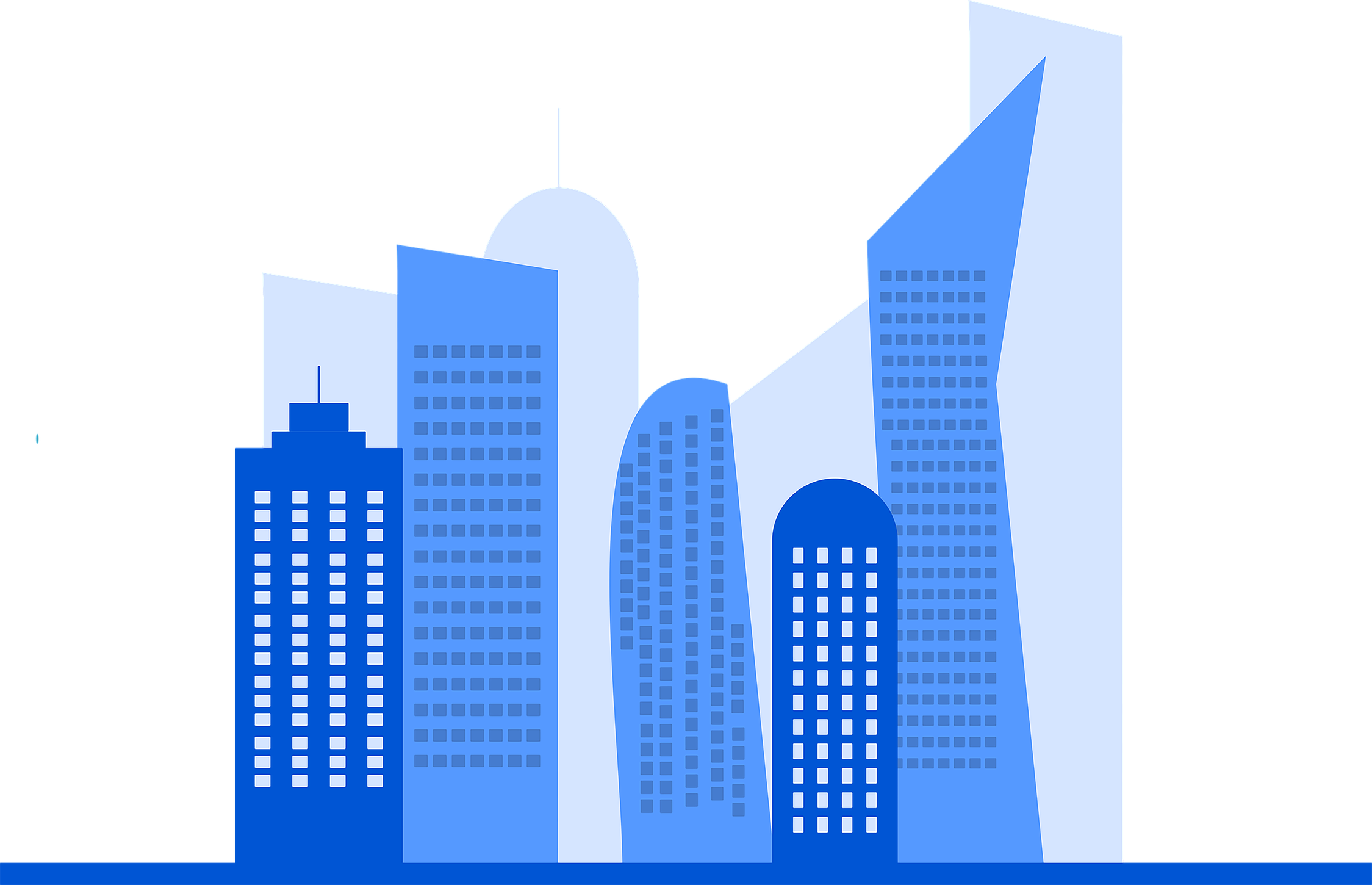

Supplement: S10 Fig — We cropped and modified the image to the components of Fig 2. Image URL: https://pixabay.com/illustrations/architecture-buildings-skyscrapers-5594350/ Image by andrezin_ce from Pixabay. Pixabay License: Free for commercial use. No attribution required. You can make modifications to content from Pixabay. (TIF) [file pone.0258062.s010.tif]

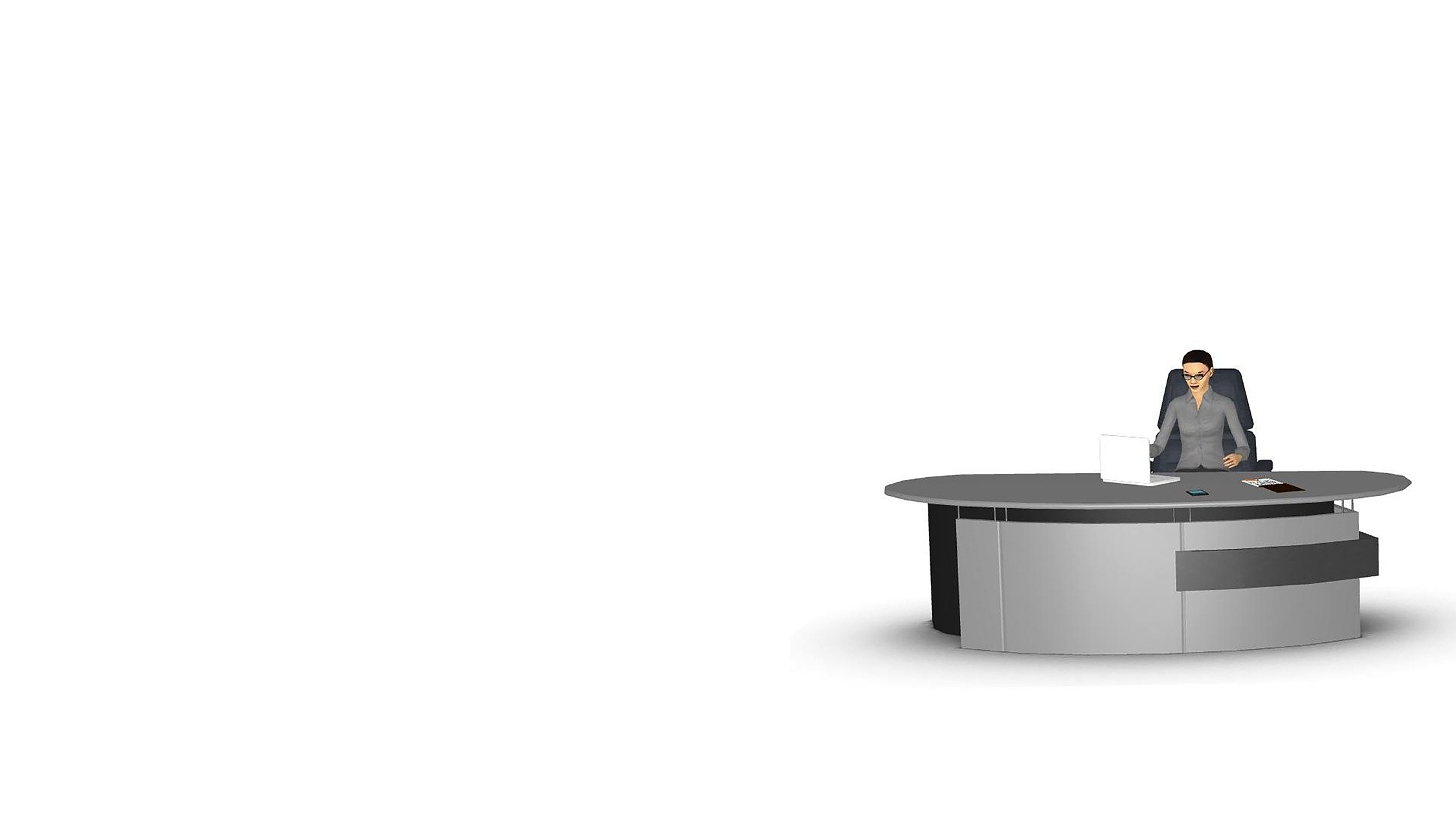

Supplement: S11 Fig — We cropped and modified the image to the components of Fig 2. Image URL: https://pixabay.com/illustrations/woman-business-desk-office-3132627/ Image by Sabrina Young from Pixabay. Pixabay License: Free for commercial use. No attribution required. You can make modifications to content from Pixabay. (TIF) [file pone.0258062.s011.tif]
