# Supplementary material for: An environmental monitoring data sharing scheme based on attribute encryption in cloud-fog computing
Source: PLoS One. 2021 Sep 30;16(9):e0258062. doi: 10.1371/journal.pone.0258062 (PMC8483347; doi:10.1371/journal.pone.0258062)
Supplement: S1 Appendix — (PDF) [file pone.0258062.s012.pdf]

**S1 Appendix. Confidentiality.**

**Theorem 1.** If the DBDH problem is difficult, and the attacker's challenge access policy is $T_a$, our scheme is IND-CPA secure, and no attacker can choose plaintext to break our scheme in polynomial time.

**Proof:** If there is a probabilistic polynomial time adversary $\mathcal{A}$ that can selectively break our proposal with a non-negligible advantage $\varepsilon$, and his challenge access policy is $T_a$, then a probabilistic polynomial time algorithm can be constructed so that challenger $\mathcal{B}$ can break the DBDH problem with a non-negligible advantage.

- **Initialization.** Challenger $\mathcal{B}$ takes DBDH challenge parameter $Q = (g, g^a, g^b, g^c)$ and $Y$ as input. Before initialization, attacker $\mathcal{A}$ selects the challenge $T_a$ and sends it to challenger $\mathcal{B}$.

- **Setup.** Challenger $\mathcal{B}$ simulates global establishment and attribute authority establishment to initialize parameters. $\mathcal{B}$ randomly selects $\beta' \in Z_p$, let $\beta = \beta' + b$, gets the public parameter $params = \{g, h, g^a, g^b, e(g,g)^{a\beta}, S, A, H_1, H_2, H_3\}$. For all $s_{a_i,k} \in S$, $\mathcal{B}$ randomly selects $q \in Z_p$, if $s_{a_i,k} \in S_{T_a}$, then makes $q_{a_i,k} = a\tilde{q}_{a_i,k}^{-1}$ and calculates $P_{a_i,k} = g^{a\tilde{q}_{a_i,k}^{-1} \cdot t_{a_i}}$; otherwise $q_{a_i,k} = \tilde{q}_{a_i,k}$, makes $P_{a_i,k} = g^{\tilde{q}_{a_i,k} \cdot t_{a_i}}$, calculates and set APK as $APK = \{APK_{a_i}\}_{\forall a_i \in A} = \{g^{t_{a_i}}, \{P_{a_i,k}\}_{1 \leq k \leq |S_{a_i}|}\}$. Challenger $\mathcal{B}$ sends $Params$ and $APK$ of the attribute authority to attacker $\mathcal{A}$.

- **Phase 1.** The attacker $\mathcal{A}$ submits attribute set $S_c \subseteq S$ to $\mathcal{B}$ to request the corresponding private key. The restriction requirement of the attribute set $S_c$ should not meet the access policy $T_a$. This scheme uses two organizations, the attribute authority AA and the environmental protection agency EPA to calculate and generate the user's private key. Challenger $\mathcal{B}$ responds to attacker $\mathcal{A}$'s private key request after $\mathcal{A}$ sends a request, $\mathcal{B}$ chooses a random number $\gamma' \in Z_p$, calculates $\gamma = \gamma' - b$ and sets $D_1 = g^{a\gamma'} h^{\theta}$, $D_2 = g^{a\theta}$, $D = g^{a(\beta+\gamma)} = g^{a(\beta'+b+\gamma'-b)} = g^{a(\beta'+\gamma')}$. For the attribute $s_{a_i,k}$ in $S_c$, if $s_{a_i,k} \in S_{T_a}$, challenger $\mathcal{B}$ calculates $D_{a_i,k} = g^{a^{-1}\tilde{q}_{a_i,k} \cdot \gamma' \cdot a t_{a_i}{}^{-1}} = g^{a^{-1} \cdot a q_{a_i,k}{}^{-1} \cdot \gamma' \cdot a t_{a_i}{}^{-1}} = g^{q_{a_i,k}{}^{-1} \cdot \gamma' \cdot a t_{a_i}{}^{-1}}$. Otherwise, $D_{a_i,k} = g^{a\gamma' \tilde{q}_{a_i,k}^{-1} \cdot t_{a_i}{}^{-1}} = g^{a\gamma' q_{a_i,k}{}^{-1} \cdot t_{a_i}{}^{-1}}$. Finally, challenger $\mathcal{B}$ transmits the private key to attacker $\mathcal{A}$, the private key $SK = \{D, D_1, D_2, \{D_{a_i,k}\}_{s_{a_i,k} \in S_c}\}$.

- **Challenge.** Attacker $\mathcal{A}$ transmits two equal-length plaintexts $m_1$ and $m_2$ to challenger $\mathcal{B}$. Challenger $\mathcal{B}$ sends $T_a$ to the FN after receives the plaintext. The FN selects a random number $v \in Z_p$ and assigns $s_{a_i,k} \in S_{T_a}$ a secret value $W_{a_i,k}$ according to $T_a$, and then the fog node sends a partial ciphertext $CT'$ to

challenger $\mathcal{B}$ after encryption, and the $CT'$ set as
$CT' = \{T_a, C_1 = g^v, C_2 = h^v, \{C_{a_i,k} = g^{q_{a_i,k} t_{a_i} W_{a_i,k}}\}_{s_{a_i,k} \in S_{T_a}}\}$.
Challenger $\mathcal{B}$ chooses a random number $\psi \in \{0,1\}$, encrypts $m_\psi$ to obtain $C = SE_{DK}(m_\psi)$, and calculates
$C_3 = m_\sigma \cdot e(g,g)^{a(\beta'+b)c} = m_\sigma \cdot e(g,g)^{a\beta' c} \cdot e(g,g)^{abc}$, $C' = g^c$, ${C_1}' = g^v \cdot g^c$, ${C_2}' = h^v \cdot h^c$. $\mathcal{B}$ sends the challenge cipher
$CT_\psi = \{T_a, C, C_3, C', {C_1}', {C_2}', \{C_{a_i,k}\}_{s_{a_i,k} \in S_{T_a}}\}$ to attacker $\mathcal{A}$.

- **Phase 2.** Same as Phase 1.

- **Guess.** Attacker $\mathcal{A}$ gets a guess about $\psi' \in \{0,1\}$, if $\psi' = \psi$, challenger $\mathcal{B}$ outputs 0 to represent $Y = e(g,g)^{abc}$; if $\psi' \neq \psi$, challenger $\mathcal{B}$ outputs 1 to indicate that $Y$ is the random number $y$ in the group $G_Y$. If $Y = e(g,g)^{abc}$, his advantage for attacker $\mathcal{A}$ in this case is $\varepsilon$ and $pr[B(g, g^a, g^b, g^c, Y = e(g,g)^{abc}) = 0] = \frac{1}{2} + \varepsilon$. If $Y = y$, this situation $CT_\sigma$ is completely random for the attacker $\mathcal{A}$, so $pr[B(g, g^a, g^b, g^c, Y = y) = 0] = \frac{1}{2}$. And there is:

$$\left| \begin{array}{l} pr[B(g, g^a, g^b, g^c, e(g,g)^{abc}) = 0] \\ -pr[B(g, g^a, g^b, g^c, y) = 0] \end{array} \right| = \frac{1}{2} + \varepsilon - \frac{1}{2} = \varepsilon$$

That is to say, challenger $\mathcal{B}$ can solve the DBDH problem with a non-negligible advantage, but this problem has been proven to be difficult, so the assumption that attacker $\mathcal{A}$ can choose to attack this scheme in plaintext with a non-negligible advantage $\varepsilon$ does not hold, so it proves that our scheme is IND-CPA secure, and comply with the confidentiality characteristics.
