# Supplementary material for: An environmental monitoring data sharing scheme based on attribute encryption in cloud-fog computing
Source: PLoS One. 2021 Sep 30;16(9):e0258062. doi: 10.1371/journal.pone.0258062 (PMC8483347; doi:10.1371/journal.pone.0258062)
Supplement: S2 Appendix — (PDF) [file pone.0258062.s013.pdf]

S2 Appendix. Unforgeability.

Theorem 2. If the CDH difficult problem is true, the digital signature proposed by our scheme can resist the existence of forgery attacks under adaptive selection message attacks, in other words, there is no attacker to break this proposal with a non-negligible advantage. The signature of our proposal cannot be forged.

Proof. If there is a polynomial-time adversary $\mathcal{F}$ that breaks the proposed scheme with a non-negligible advantage under the adaptive selection message attack, then a polynomial-time algorithm can be constructed to enable challenger $\mathcal{C}$ to break the CDH problem with a non-negligible advantage, that is to say, $\mathcal{C}$ knows $P_1 = ag$ and $P_2 = bg$, And finally abg can be output.

- **Initialization.** Challenger $\mathcal{C}$ runs the global establishment phase, makes the sign public key as $P_0 = MK \cdot g$, generates system parameter $Params$, and sends them to $\mathcal{F}$, where $\mathcal{F}$ does not know MK. The lists L_1, L_2, L_3 correspond to the query results of H_1, H_2, H_3 respectively, and the lists P^{list}, S^{list} correspond to the query of some private keys and the query of the secret value.

- **Query phase.** $\mathcal{F}$ must first inquire about the identity id' before it can be used for other inquiries. $\mathcal{F}$ cannot use the result of the signature query to perform the de-signature query.
  Query : When $\mathcal{F}$ inputs data (id', rg), $\mathcal{C}$ sets the list L_1 in this format, if there is a corresponding record $(id', rg, W_{id'})$ in L_1, then outputs $W_{id'}$, otherwise $\mathcal{C}$ chooses a random number $W_{id'} \in Z_p$, adds $(id', rg, W_{id'})$ to the list L_1 and returns $W_{id'}$ to $\mathcal{F}$.
  Query : When $\mathcal{F}$ inputs data (id', l, rg, P_0), $\mathcal{C}$ sets the list L_2 in this format, if there is a corresponding record $(id', l, rg, P_0, \sigma_1)$ in L_2, then outputs σ_1, otherwise $\mathcal{C}$ chooses a random number $\sigma_1 \in Z_p$, adds $(id', l, rg, P_0, \sigma_1)$ to the list L_2 and returns σ_1 to $\mathcal{F}$.

Query : When $\mathcal{F}$ inputs data (M, id', l, rg, P_0), $\mathcal{C}$ sets the list L_3 in this format, if there is a corresponding record $(M, id', l, rg, P_0, \sigma_2, c)$ in L_3, then returns σ_2, otherwise $\mathcal{C}$ randomly selects $c \in Z_p$ and calculates $\sigma_2 = P_2 + cg$, adds $(M, id', l, rg, P_0, \sigma_2, c)$ to the list L_3, and returns σ_2 to $\mathcal{F}$.

- **Private key and secret value query.** When $\mathcal{F}$ asks for partial private key id', $\mathcal{C}$ selects two random numbers $a_{id'}, b_{id'} \in Z_p$ and lets $r \leftarrow a_{id'}$, $rg \leftarrow a_{id'} \cdot g$, $W_{id'} = H_1(id', rg) \leftarrow b_{id'}$, calculates $Q = r + W_{id'} \cdot MK$. Finally, $\mathcal{C}$ write $(id', rg, W_{id'})$ into the list L_1, and write (id', Q, rg) into the list P^{list}. $\mathcal{F}$ asks for the secret value of id', if $id' = id$, $\mathcal{C}$ randomly selects a number $\mu \in Z_p$ and calculates $l = \mu g$, return μ to $\mathcal{F}$ and write (id', μ, l) into P^{list}; if $id' \neq id$, let $l \leftarrow P_1$, then write $(id', \perp, l)$ into the list S^{list}.

- **Signature query.** $\mathcal{F}$ asks for the signature of (id', M), and $\mathcal{C}$ finds partial private key (id', Q, rg) and secret value (id', μ, l) in the list P^{list}, S^{list}. Execute the query H_2 by $\mathcal{C}$ to get the $\sigma_2 = H_2(M, id', l, rg, P_0)$ corresponding $(M, id', l, rg, P_0, \sigma_2, c)$ in list L_3. Let $\sigma_2 = cg$, then $\mathcal{C}$ returns the signature $\sigma' = c(\sigma_1 \cdot l + rg + W_{id'} \cdot P_0)$.
  Finally, $\mathcal{F}$ outputs a valid signature (id', M, σ'), $\mathcal{C}$ calculates $\sigma_2 = H_2(M, id', l, rg, P_0)$, where (l, rg) is the original signature public key of id', and then verifies whether the equation $e(\sigma', g) = e(\sigma_2, (\sigma_1 \cdot l + rg + W_{id'} \cdot P_0))$ is true, if not, $\mathcal{C}$ immediately reports an error and stops execution; otherwise, obtains the equation $e(sign, g) = e(\sigma_2, (\sigma_1 \cdot l + rg + W_{id'} \cdot P_0))$. Among them $(M, id', l, rg, P_0, \sigma_2, c)$ are all elements in the list L_3.

- **Forgery.** $\mathcal{C}$ calls out the list L_1, L_2, L_3 and P^{list}, S^{list} then calculates $\sigma_2 = bg + cg$, $\sigma' = (b + c)(\sigma_1 a + r + W_{id'} \cdot MK)g$, so $\mathcal{C}$ can output $abg = (\sigma_1)^{-1} \cdot (\sigma' - c(\sigma_1 P_1 + rg + W_{id'} \cdot P_0) - (r + W_{id'} \cdot MK)P_2)$. If $\mathcal{F}$ can forge a?legitimate signature successfully, challenger $\mathcal{C}$ could solve the CDH problem. Still, the CDH problem has been proved difficult, so the assumption that adversary $\mathcal{F}$ can break the scheme with a non-negligible advantage does not hold. Therefore, our proposal is unforgeable under the selective message attack.
